# Kuanxiong aerosol protects against coronary microvascular dysfunction through improving angiogenesis via HIF-1α-ITGAX axis

**DOI:** 10.3389/fphar.2026.1881934

**Published:** 2026-08-13

**Authors:** Yuanqing Jiang, Kai Qian, Beibo Li, Mengmeng Xu, Pengfei OuYang, Yangyang Zhu, Jiaxu Wu, Xiaoying Shi, Yi Luo, Yao Xie, Meixiang Xiang

**Affiliations:** 1 Department of Cardiology, The Second Affiliated Hospital, School of Medicine, Zhejiang University; State Key Laboratory of Transvascular Implantation Devices; Heart Regeneration and Repair Key Laboratory of Zhejiang Province, Hangzhou, China; 2 Zhejiang Provincial Key Research Institute of Traditional Chinese Medicine for the Prevention and Treatment of Cardiovascular and Cerebrovascular Diseases, Hangzhou, China

**Keywords:** coronary microvascular dysfunction, diabetes mellitus, Kuanxiong aerosol, angiogenesis, HIF-1α-ITGAX axis, VEGF-FAK pathway

## Abstract

**Background:**

Coronary microvascular dysfunction (CMVD) frequently complicates diabetes mellitus and serves as a major driver of cardiovascular morbidity, underscoring the urgent need for innovative therapeutic strategies. Kuanxiong aerosol (KX), a patented botanical formulation of four volatile oils widely used for angina, possesses established vasoprotective and hemodynamic benefits. However, its efficacy and mechanisms in CMVD remain uncharacterized.

**Methods:**

To evaluate the therapeutic potential of KX, both preventive and therapeutic treatment regimens were administered to a db/db mouse model of diabetic CMVD. In vivo therapeutic effects were comprehensively assessed using echocardiography and histopathological analyses. Microvascular perfusion and endothelial barrier function were quantitatively evaluated via Lectin-FITC perfusion and permeability assays.These findings were further corroborated by bulk RNA sequencing, immunofluorescence, and cellular functional assays, while bioinformatics and biochemical analyses systematically revealed the molecular targets and pathways.

**Results:**

KX restored coronary microvascular structure and function, promoting neovascularization and conferring both preventive and therapeutic benefits against CMVD in db/db mice. Mechanistically, RNA sequencing and biochemical analyses revealed that KX stabilizes HIF-1α against PHD2-mediated proteasomal degradation. This stabilization enhances HIF-1α transcriptional activity to upregulate ITGAX, which subsequently triggers the VEGF-FAK signaling pathway to drive endothelial proliferation, angiogenesis and tight junction formation. Consequently, the improved microvascular function and stabilized nascent vascular network restore myocardial blood supply, thereby rescuing CMVD.

**Conclusion:**

KX exerts its therapeutic effects through modulation of the HIF-1α–ITGAX axis, thereby promoting angiogenesis and restoring microvascular integrity and perfusion. These findings establish KX as a promising therapeutic candidate for CMVD and diabetic cardiomyopathy.

## 1 Introduction

Diabetic cardiomyopathy (DCM) is increasingly recognized as a distinct cardiovascular complication of diabetes mellitus, characterized by diastolic dysfunction, pathological cardiac hypertrophy, and excessive fibrotic deposition independent of overt coronary artery disease or hypertension. As a leading contributor to heart failure and diabetes-related mortality worldwide, DCM poses a formidable clinical challenge (Shou et al., 2024; Horton and Barrett, 2021). Clinically, diabetic patients commonly present with microvascular angina, also termed ischemia with no obstructive coronary artery disease (INOCA) (Zhao et al., 2022). This symptomatic manifestation underscores the pivotal role of coronary microvascular dysfunction (CMVD) as a central driver of DCM pathogenesis. Notably, intensive glycemic control offers limited efficacy in mitigating angina and preventing DCM progression, highlighting a critical therapeutic void in targeting CMVD.

CMVD is functionally defined by endothelial dysfunction, reduced myocardial perfusion reserve, and impaired angiogenesis, all of which accelerate cardiac dysfunction (Qiu et al., 2022; Wang et al., 2022). Under chronic hyperglycemia, insulin resistance, and metabolic stress, the coronary microcirculation undergoes severe alterations—including defective endothelial sprouting and heightened vascular permeability (Horton and Barrett, 2021). Crucially, these microvascular defects precede the onset of overt ventricular remodeling and myocardial fibrosis, thereby highlighting the importance of microvascular structural and functional integrity in preserving cardiac function (Qiu et al., 2022). Given the multifactorial nature of diabetic cardiovascular complications, multi-component, multi-targeted therapeutic strategies have garnered substantial interest.

Kuanxiong aerosol (KX), a patented traditional Chinese medicine developed in accordance with the Chinese Pharmacopoeia based on aromatic and warming-up principles, is formulated as a volatile oil blend derived from four distinct botanical sources-*Santalum album* L. (sandalwood), *Alpinia officinarum* Hance (galangal), *Asarum sieboldii* Miq. (asarum) and *Piper longum* L. (long pepper)-closely integrated with borneol (Chen et al., 2025). This formulation has demonstrated profound vasoprotective and hemodynamic-stabilizing effects in patients with angina (Zhang et al., 2021; Zhuang et al., 2020; Qian et al., 2026). Markedly, a recent randomized controlled trial indicated that KX significantly improved perioperative coronary microcirculation in unstable angina patients undergoing elective percutaneous coronary intervention (PCI), as evidenced by a lower post-PCI index of microcirculatory resistance (IMR) (Liu et al., 2025). These clinical insights strongly suggest that KX may serve as a potent therapeutic agent for microvascular diseases. However, its role and underlying mechanism in CMVD associated with diabetic cardiomyopathy remain unknown.

To address this gap, the present study investigated the therapeutic potential and mechanistic framework of KX in *db/db* mice, a well-established model of type 2 diabetes-related cardiovascular complications (Ferreira et al., 2020; Coleman, 1982; Bu et al., 2019; Adingupu et al., 2019; Su et al., 2020). We show that KX protects against CMVD by restoring coronary perfusion and preserving microvascular structural integrity. Mechanistically, our findings reveal that KX exerts its therapeutic benefits by stabilizing and activating the HIF-1α-ITGAX signaling axis. These findings establish KX as a promising and clinically relevant therapeutic candidate for both CMVD and diabetic cardiomyopathy.

## 2 Materials and methods

### 2.1 Preparation and formulation of Kuanxiong aerosol

Kuanxiong aerosol (KX, Batch No. 22408006, manufactured on 20 August 2024; expiry: July 2027) was provided by Zhejiang Sukean Pharmaceutical Co., Ltd. (Zhejiang, China). This commercial formulation is certified by the National Medical Products Administration (NMPA, China) under approval number Z20163023.

The KX formulation was prepared strictly adhering to the official standards documented in the Pharmacopoeia of the People’s Republic of China (2020 Edition, Volume I, pp. 1524–1526), following methodology benchmarked against previous studies (Qian et al., 2026). Briefly, KX comprises a validated blend of borneol and four botanical volatile oils extracted from *S. album* L. (sandalwood), *A. officinarum* Hance (galangal), *Asarum sieboldii* Miq. (asarum) and *P. longum* L. (long pepper) with corresponding Certificates of Analysis (CoA) provided in Supplementary Figure S13. All botanical sourcing, collection, and processing complied fully with the Nagoya Protocol, CITES, and relevant national/international phytosanitary regulations.

To harvest the volatile intermediates, raw botanical materials were appropriately crushed or sliced, submerged in distilled water (0.5–2 times the biomass weight) and hydro-distilled using a clevenger-type apparatus under continuous boiling for herb-specific durations: at least 50 h for sandalwood, 7 h for long pepper and galangal and 5 h for asarum. Subsequently, the extracted oils of A. sieboldii (23 mL), S. album (70 mL), A. officinarum (32 mL), and P. longum (25 mL) were mixed on a 40 °C water bath. Upon complete dissolution of solid borneol (22.5 g) under gentle agitation, the mixture was brought up to 625 mL with anhydrous ethanol and clarified via filtration. The ultimate stock solution contained 11.2% v/v sandalwood oil, 4.0% v/v long pepper oil, 5.12% v/v galangal oil, 3.68% v/v asarum oil, and 36 mg/mL borneol, and was stored light-protected at room temperature.

To ensure batch-to-batch consistency, KX was prepared strictly according to the official formulation. The gas chromatography-flame ionization detection (GC-FID) fingerprinting profile of this batch, overlaid with the reference KX product, is presented with characteristic peaks and identified compounds (Supplementary Figure S9; Table 1). The official COA and multi-batch overlaid GC-FID fingerprints of KX are also provided, with cross-batch consistency evaluated by orthogonal analysis (Supplementary Figure S10; Table 2). Additionally, two major diagnostic markers of KX, namely, methyleugenol and borneol, were further quantified by quantitative proton nuclear magnetic resonance (q^1^H NMR), with representative spectra and quantitative data provided (Supplementary Figure S11; Table 3).

**TABLE 1:** Twelve chemical markers of Kuanxiong aerosol (KX) specified by the Chinese Pharmacopoeia.

Peak No.	Primary source	Chemical compound
1	*Alpinia officinarum* Hance	β-Pinene
2	*Alpinia officinarum* Hance	Eucalyptol
3	*Asarum sieboldii* Miq.	2,4-Cycloheptadien-1-one,2,6,6-trimethyl
4	*Borneolum Syntheticum*	DL-isoborneol
5	*Borneolum Syntheticum*	Borneol
6	*Asarum sieboldii* Miq.	1,3-Benzodioxole,5-(2-propenyl)
7	*Asarum sieboldii* Miq.	Methyleugenol
8	*Piper longum* L.	1,6-Cyclodecadiene,1-methyl-5-methylene8-(1-methylethyl)-, [s-(E,E)]
9	*Asarum sieboldii* Miq.	Benzene,1,2,3-trimethoxy-5-(2-propenyl)
10	*Santalum album* L.	Santalol,cis,alpha
11	*Santalum album* L.	α-Bisabolol
12	*Santalum album* L.	6-(p-Toly)-2-methyl-2-heptenol

**TABLE 2:** Similarity evaluation of chromatographic fingerprints across multi-batches of KX.

Batch number	22407013	22408006	22409013	22410005	22411008	22412005	Reference fingerprint
22407013	1.000	1.000	1.000	1.000	1.000	1.000	1.000
22408006	1.000	1.000	1.000	1.000	1.000	1.000	1.000
22409013	1.000	1.000	1.000	1.000	1.000	1.000	1.000
22410005	1.000	1.000	1.000	1.000	1.000	1.000	1.000
22411008	1.000	1.000	1.000	1.000	1.000	1.000	1.000
22412005	1.000	1.000	1.000	1.000	1.000	1.000	1.000
Reference fingerprint	1.000	1.000	1.000	1.000	1.000	1.000	1.000

**TABLE 3:** Quantification of methyleugenol and borneol in multi-batches of KX via q^1^H NMR.

Batch No.	Methyleugenol (mg/mL)	Borneol (mg/mL)
22407013	8.19311346	25.00849
22408006	11.48370552	26.22546
22409013	9.827287259	23.08814
22410005	11.40346656	23.36042
22411008	9.447542448	20.89553
22412005	9.545259209	19.29764

### 2.2 GC-MS quantification of Kuanxiong aerosol

Headspace gas chromatography-mass spectrometry (HS-GC-MS) was employed to identify the chemical components of KX as described previously. Briefly, analyses were performed on an Agilent 7890B gas chromatograph coupled with a 5977B MSD mass spectrometer, using an HP-5MS capillary column (30 m × 0.25 mm i.d., 0.25 μm film thickness; Agilent 19091S-433). The oven temperature was programmed from 40 °C (held 3 min) to 300 °C at 5 °C/min (held 3 min; maximum 325 °C). A 1 µL sample was injected in split mode (280 °C, 40:1) via the front SS inlet, with helium as carrier gas (1.0 mL/min). The rear SS inlet was operated in pulsed splitless mode (25 psi, cutoff 0.5 min). A gas sampling valve (GSV, 1 mL loop) was used with a filling and injection time of 0.5 min. Data acquisition was performed at 50 Hz (solvent delay 3 min), with ion source and quadrupole temperatures of 230 °C and 150 °C, respectively. The complete compound list and relative abundance of KX via GC-MS are provided (Supplementary Table S1; Supplementary Figure S12A), and representative fingerprint profiles obtained by q^1^H NMR and liquid chromatography-mass spectrometry (LC-MS), along with major marker compounds identified by each analytical platform, are presented (Supplementary Figures S12B,C; Tables 4–6).

**TABLE 4:** Major characteristic constituents of KX identified via HS-GC-MS.

Target compound	RT	Area%-T
β-Pinene	10.6543	0.76
Eucalyptol	12.4371	13.99
2,6,6-Trimethyl-2,4-cycloheptadien-1-one	16.1975	1.84
Methyleugenol	23.2299	8.94
Humulene	24.5257	1.21
Elemicin	26.9484	0.80
α-Santalol	29.8175	0.45

**TABLE 5:** Proton assignments and diagnostic integration signals for the eight markers of KX in q^1^H NMR analysis.

Marker	Chemical shift (δ, ppm)	Multiplicity	Proton assignment
β-Pinene	4.59–4.61	s	H-1a (olefinic)
Eucalyptol	1.03, 1.24	s	H-1, H-5,6 (methyl)
Methyleugenol	6.76–6.79	s	H-6 (aromatic)
Elemicin	6.47–6.49	s	H-5,15 (aromatic)
2,6,6-Trimethyl-2,4-cycloheptadien-1-one	2.63–2.66	s	H-9 (methyl)
α-Humulene	5.57–5.65	Td (J = 7.4, 15.4 Hz)	H-14 (olefinic)
α-Santalol	1.00–1.02	s	H-14 (methyl)
Borneol	4.03–4.06	m	H-2 (methine)

**TABLE 6:** Major chemical constituents of KX identified via LC-MS.

Target compound	RT	m/z
β-Pinene	17.29	137.1325
2,6,6-Trimethyl-2,4-cycloheptadien-1-one	17.295	151.1117
Methyl eugenol	17.584	179.1067
α-Santalol	22.534	221.19
Humulene	23.725	205.1951

### 2.3 Animals and experimental design

Adult male *db/db* (Lepr^db^/^db^) and non-diabetic *db/m* littermates, together with 15-week-old male *db/db* mice, were obtained from Jiangsu GemPharmatech Co., Ltd. Animals were housed under standard conditions (12-h light/dark cycle, 22 °C–24 °C, 55%–60% humidity) with free access to food and water. The study was approved by the Institutional Animal Care and Use Committee (IACUC) of Zhejiang Provincial Key Research Institute of Traditional Chinese Medicine for the Prevention and Treatment of Cardiovascular and Cerebrovascular Diseases (Reference No. ZJSKA-IACUC-241101) and performed in accordance with relevant guidelines.

To evaluate the effect of KX on coronary microvascular disease, 8-week-old *db/db* mice were randomly divided into four groups: Saline (daily saline), KX-L (40 mg/kg KX), KX-M (80 mg/kg KX), and KX-H (160 mg/kg KX), all administered sublingually once daily. Non-diabetic *db/m* mice served as negative controls. All animals were fed a normal chow diet for 8 weeks.

To investigate the therapeutic potential of KX in the regression of CMVD, 16-week-old *db/db* mice were randomly assigned to four groups: Baseline (echocardiography at 16 weeks), Saline (sublingually daily saline for 8 weeks), KX (80 mg/kg sublingually daily for 8 weeks), and Empagliflozin (10 mg/kg intragastrically daily for 8 weeks; MCE, China, #HY15409). All mice were fed a normal chow diet. At the end of the treatment period, all mice were euthanized via isoflurane inhalation (up to 5% concentration) for terminal blood and tissue collection.

### 2.4 Transthoracic echocardiography

Mice were anesthetized with 2%–2.5% isoflurane (RWD, China, #R510-22-10) and maintained with 1.5% isoflurane via a nose cone during echocardiography. Transthoracic echocardiography was performed using a Vevo2100 system (VisualSonics) with a 40-MHz transducer. M-mode and color Doppler images were recorded, and left ventricular ejection fraction, fractional shortening, and left ventricular mass were calculated using VevoLAB software (v2.2.3).

Coronary flow velocity reserve (CFVR) was assessed as described (Adingupu et al., 2019; Su et al., 2020). Isoflurane was reduced to 1% to establish baseline coronary flow; stable velocity signals in the left coronary artery were collected for 3 min. Isoflurane was then increased to 2.5% to induce hyperemia, and signals were recorded for up to 4 min. CFVR was calculated as the ratio of peak diastolic flow velocities during hyperemia to baseline (2.5%/1% isoflurane).

### 2.5 Serum biochemical assays

Blood was collected via retro-orbital bleeding and clotted for 30 min at room temperature. Serum was obtained by centrifugation at 3,000 × *g* for 10 min at 4 °C. Serum levels of triglyceride (TG) and total cholesterol (TC) were measured using commercial kits (Abcam, USA, #ab65336 and #ab282928). B-type natriuretic peptide (BNP) was determined by ELISA (Cusabio, China, #CSB-E07971m). Liver function markers, including alanine aminotransferase (ALT) and aspartate aminotransferase (AST), and kidney function markers, including blood urea nitrogen (BUN), creatinine (CREA) and uric acid (UA), were assessed using commercial kits from mlbio (mlbio, China, #mlC50573-2, #mlC50572-2, #ml037577-2, #ml037580-2, #ml092696, respectively) according to the manufacturer’s instructions.

### 2.6 The intraperitoneal glucose tolerance and insulin tolerance test

Intraperitoneal glucose tolerance test (IGTT) and insulin tolerance test (ITT) were performed as previously described with slight modifications (Qin et al., 2026). Blood glucose levels were measured using an Accu-Chek Guide glucometer (Roche, Switzerland) with corresponding test strips. For the IGTT, *db/m* and *db/db* mice were fasted overnight and then subjected to an intraperitoneal injection of glucose (2 g/kg). Blood samples were collected from the tail vein at baseline (0), 15, 30, 60, and 90 min post-injection for blood glucose measurement. For the ITT, overnight-fasted mice received an intraperitoneal injection of insulin (1 IU/kg body weight), and blood glucose was measured from tail blood at 0, 15, 30, 60, 90, 120, and 150 min post-injection using the same glucometer.

### 2.7 Histopathological analysis

Mouse hearts were harvested after euthanasia, weighed, and heart weight was normalized to tibial length to assess cardiac hypertrophy. Tissues were fixed in 4% paraformaldehyde (PFA; Solarbio, China, #P1110), embedded in paraffin and sectioned (5 µm). To evaluate cardiomyocyte size, sections were stained with hematoxylin-eosin (H&E; Solarbio, China, #G1120) and wheat germ agglutinin (WGA; MCE, China, #HYNP163A). Quantification of cardiomyocyte cross-sectional area was performed using ImageJ software. To evaluate the extent of cardiac fibrosis and collagen deposition, Masson’s trichrome staining and Picrosirius Red staining were performed as previously described (Xing et al., 2024). The cross-sections of the heart tissue were stained with Masson’s trichrome (Solarbio, China, #G1340) and Picrosirius Red (PSR; Solarbio, China, #G1470). Collagen volume fraction was quantified using ImageJ software and expressed as a percentage of the total tissue area.

### 2.8 Cell culture and treatment

Human cardiac microvascular endothelial cells (HCMECs, Lonza, Switzerland, #CC-7030) were cultured in endothelial cell medium (ECM, ScienCell, USA, #1001) supplemented with growth supplements, 5% FBS and 1% penicillin-streptomycin at 37 °C in 5% CO_2_. Cells from passages 3 to 10 were used.

High glucose and high fatty acid (HGHF) treatments were applied as described in a previous study (Li et al., 2023). After reaching 90% confluence, the HCMECs were placed in a medium containing 25 mM glucose and 500 μM free fatty acids for 72 h. The mixture comprised bovine serum albumin- (BSA)-conjugated palmitic acid (PA) and oleic acid (OA) in a ratio of 1:2 (Shanghai Siduorui Biotechnology Service, China, #FFA01). Control cells received 19.5 mM mannitol (MCE, China; #HYN0378) to exclude osmotic effects. The HGHF condition (25 mM glucose plus 500 μM free fatty acids, PA: OA = 1:2) was selected to recapitulate the combined hyperglycemic and dyslipidemic milieu characteristic of type 2 diabetes *in vitro*, consistent with established protocols for diabetic cardiac microvascular injury modeling (Chen Y. et al., 2024; Chen Z. et al., 2024).

For KX treatment, the aerosol was discharged into a sterile pre-chilled (−20 °C) glass vial, equilibrated at room temperature, and incubated at 37 °C for 10 min (unsealed) to ensure complete evaporation of the propellant tetrafluoroethane. The resulting volatile oil was dissolved in DMSO and diluted in complete ECM to the working concentration. An equivalent volume of DMSO-supplemented medium served as the vehicle control (final DMSO < 0.1% v/v in all groups). Sterile filtration was omitted to prevent membrane adsorption of lipophilic constituents, as sterility was assured by commercial manufacturing conditions and laminar flow hood operation. The identical Chinese Pharmacopoeia-certified batch (Batch No. 22408006) was used for all *in vivo* and *in vitro* experiments.

For the Hypoxia-inducible factor-1α (HIF-1α) protein stability assay, 100 μg/mL CHX (MCE, China; # HY-12320) was treated to cells for the indicated durations. DMSO-treated cells served as the vehicle control. For signaling pathway analysis, cells transfected with siRNA were subjected to HGHF treatment or DMSO vehicle control, serum-starved for 4 h prior to harvest, and collected at the indicated time points as described in the figure legends. To assess hydroxy-HIF-1α expression and prolyl hydroxylase (PHD) enzymatic activity, HCMECs were subjected to a 48-h HGHF condition, with KX, DMSO, or the PHD inhibitor dimethyloxalylglycine (DMOG; 1 mM) for the last 6 h, with 20 μM MG132 introduced for 2 h prior to harvest to prevent the proteasomal degradation of HIF-1α.

### 2.9 siRNA transfection

HCMECs were cultured in 6-well plates until they reached 50%–70% confluence with 24 h HGHF treatment, and then cells were transfected with 50 nM negative control siRNA or siRNAs against HIF-1α (OriGene Technologies, China, #SR320469), and Integrin subunit alpha X (ITGAX, OriGene Technologies, China, #SR302469) by using Lipofectamine RNAiMAX (Invitrogen, USA, #13778150) with Opti-MEM medium for 6 h incubation. After that, the ECM medium was added into the cell and cultured for 72 h, the cells were collected for analysis.

### 2.10 Lactate dehydrogenase (LDH) release assay

Cell membrane integrity was evaluated by a LDH release assay using an LDH cytotoxicity assay kit (Beyotime Biotechnology, China, #C0016) according to the manufacturer’s instructions. Briefly, cells were seeded in 96-well plates and subjected to the indicated treatments. Culture supernatants were collected following centrifugation (400 × *g*, 5 min). The working reagent was then mixed with supernatants and incubated at room temperature in the dark for 30 min. LDH activity was measured spectrophotometrically at 490 nm. Maximum LDH release was determined by treating cells with the provided lysis buffer for 1 h prior to supernatant collection. LDH release (%) was calculated by dividing the background-corrected optical density of the sample by that of the maximum release control.

### 2.11 Cell viability assay

Cell viability was measured by the cell-counting kit-8 (CCK8) assay (Beyotime Biotechnology, China, #C0038) after treatment according to the instruction protocols.

### 2.12 Lectin-FITC perfusion assay and tissue permeability assay

Mice received a tail-vein injection of 100 μL lectin-FITC (0.1 mg/mL; Sigma-Aldrich, USA, #L0401). After 15 min, hearts were collected, embedded in OCT (Sakura, USA, #4583), frozen, and cryosectioned (5 µm). Sections were stained for CD31 and observed under a confocal microscope for lectin/CD31 colocalization.

### 2.13 Bulk RNA sequencing

Total RNA was extracted from HCMECs using TRIzol reagent (Invitrogen, USA, #15596026CN) according to the manufacturer’s instructions. RNA sequencing was performed by LC-Bio Technologies (Hangzhou, China) with three biological replicates per group. Gene expression levels were quantified as fragments per kilobase of exon per million mapped reads (FPKM) using HTSeq-count. Differentially expressed genes (DEGs) were identified using DESeq2, with thresholds set at a false discovery rate (FDR) < 0.05 and |fold change| ≥ 1.5. Volcano plots, heatmaps, and gene set enrichment analysis (GSEA) were generated using OmicStudio (https://www.omicstudio.cn/cell). Raw sequencing data have been deposited in the NCBI database under BioProject accession number PRJNA1472894. The raw sequencing data have been deposited in NCBI under BioProject accession number PRJNA1472894 (https://www.ncbi.nlm.nih.gov/bioproject/PRJNA1472894/).

### 2.14 RNA isolation and qPCR assays

Total RNA was extracted using a Universal RNA Extraction Kit (Accurate Biology, China, #AG21024) and reverse transcribed into cDNA using the PrimeScript™ RT Master Mix Kit (Accurate Biology, China, #AG11706). qPCR was performed on a 7500 Fast Real-Time PCR System using SYBR Green Premix (Accurate Biology, China, #AG11740). Relative mRNA expression was calculated using the 2^−ΔΔCT^ method with β-actin as the internal control. Primer sequences are listed below:

Human ITGAX Forward (5′-3′): AGA​GCT​GTG​ATA​AGC​CAG​TTC​C; Reverse (5′-3′): AAT​TCC​TCG​AAA​GTG​AAG​TGT​GT; Human HIF-1α Forward (5′-3′): TTC​CCG​ACT​AGG​CCC​ATT​C; Reverse (5′-3′): CAG​GTA​TTC​AAG​GTC​CCA​TTT​CA; Human β-actin: Forward (5′-3′): CAT​GTA​CGT​TGC​TAT​CCA​GGC; Reverse (5′-3′): CTC​CTT​AAT​GTC​ACG​CAC​GAT.

### 2.15 Transcription factor prediction and binding motif conservation analysis

Potential transcription factors (TFs) regulating ITGAX and their binding motifs were predicted using the hTFtarget database (https://guolab.wchscu.cn/hTFtarget/#!/), the AnimalTFDB database (http://bioinfo.life.hust.edu.cn/AnimalTFDB#!/) and the JASPAR Database (https://jaspar.genereg.net/). To assess binding motif conservation, the nucleotide sequences of the ITGAX promoter across multiple species were retrieved from the NCBI database and aligned using ClustalW. Conserved regions were visualized as sequence logos.

### 2.16 *In vivo* vascular permeability assay

The *db/m* and *db/db* mice received an intravenous injection of 70 kDa dextran-FITC (0.5 mg/mouse; MCE, China, #HY128868E). After 1 h, hearts and plasma were collected. The fluorescence of FITC-dextran in plasma was measured using a spectrophotometer (excitation 490 nm, emission 520 nm; Tecan, Switzerland, Infinite® M Nano). Heart sections (5 µm) were stained with VE-cadherin antibody, and dextran-FITC/VE-cadherin colocalization was visualized with an LSM900 confocal microscope (Carl Zeiss, Germany).

### 2.17 Immunofluorescence (IF) analysis

PFA-fixed cell coverslips and 5-µm sections were washed three times with PBS, permeabilized with 0.25% Triton X-100 in PBS for 10 min, and blocked for 1 h at room temperature in blocking buffer (5% horse serum, 2% BSA in PBS). Slides were incubated with primary antibody (in blocking buffer) overnight at 4 °C, then with secondary antibody for 1 h at room temperature. Nuclei were stained with DAPI (1:1,000, Solarbio, China, #D8200) for 15 min. After mounting with Antifade Mounting Medium (Thermo Fisher, USA, #S36937), images were acquired using an LSM900 confocal microscope. Mean fluorescence intensity was quantified with ImageJ. Antibodies and reagents are listed in Table 7.

**TABLE 7:** Reagents and antibodies.

Antibody	Company	Catalog No.	Application
ITGAX	CST	97585	IF: 1:250
VE-cadherin (IF for cell)	CST	2500	IF: 1:250
Ki67	Abcam	ab15580	IF: 1:250
CD31	R&D Systems	AF3628	IF: 1:250
Alexa Fluor® 594 goat anti-rabbit IgG (H + L)	Abcam	ab150080	IF: 1:500
Alexa Fluor® 488 goat anti-rabbit IgG (H + L)	Abcam	ab150077	IF: 1:500
Alexa Fluor®488 donkey anti-goat IgG (H + L)	Abcam	ab150129	IF: 1:500
VE-cadherin (IF for tissue)	BD bioscience	555289	IF: 1:250
Alexa fluor 594-conjugated donkey anti-rat IgG	Invitrogen	A21209	IF: 1:500
Bovine serum albumin	Fdbio	FD0030	IF
Horse serum	Sangon	E510006	IF
pFAK	CST	3283	WB: 1:1000
VHL	Proteintech	24756-1-AP	WB: 1:1000 IP: 1:100
K48-linkage-specific polyubiquitin	CST	8081	WB: 1:1000
Normal rabbit IgG	CST	2729	IP: 1:100
Hydroxy-HIF-1α (pro564)	CST	3434	WB: 1:1000
FAK	CST	3285	WB: 1:1000
p-VEGFR2	CST	2478	WB: 1:1000
VEGFR2	CST	2479	WB: 1:1000
ITGAX	CST	45581	WB: 1:1000
HIF-1α	CST	36169	WB: 1:1000 IP: 1: 100
Flag	Abmart	M20008	WB: 1:1000
β-Actin	CST	4967	WB: 1:1000
HRP-conjugated goat anti-mouse IgG (H + L)	ABclonal	AS003	WB: 1:1000
HRP-conjugated goat anti-rabbit IgG (H + L)	ABclonal	AS014	WB: 1:1000
FITC-conjugated anti-ITGAX antibody	eBioscience	11–0116-42	FC: 5 µL per 100 μL cells (1 × 10^6^ cells)
Mouse IgG1 kappa isotype control	eBioscience	11–4714-42	FC: 5 µL per 100 μL cells (1 × 10^6^ cells)

### 2.18 Western blotting

After PBS washing, cells were lysed in RIPA buffer (Beyotime, China, #P0013B) containing PMSF (Beyotime, China, #ST505) and phosphatase inhibitors (Roche, Switzerland, #04906837001). Protein concentration was determined using a BCA assay kit (Fdbio, China, #FD 2001). Equal amounts of protein were separated by 8%, 10% or 12% SDS-PAGE, transferred to PVDF membranes (Millipore, USA, #IPVH00010), and blocked with 5% milk in TBST for 1 h at room temperature. Membranes were incubated with primary antibodies overnight at 4 °C, followed by HRP-conjugated secondary antibodies (anti-rabbit or anti-mouse) for 1 h at room temperature. Chemiluminescence was detected using an ECL kit (Fdbio, China, #FD8020) on a ChemiDoc XRS system (Bio-Rad), and band intensities were semi-quantified with ImageJ. Antibodies are listed in Table 7.

### 2.19 Cell scratch assay

Cell scratch assay was performed following the previous study with slight modifications (Chen et al., 2026). After treatment, HCMECs were placed into 6-well plates and cultured until the cells adhered. A scratch was made using a plastic pipette tip and the cells were washed with PBS. After 48 h, cell migration was assessed by measuring the wound area under a Nikon inverted phase-contrast microscope and the area of cell migration was analyzed by using ImageJ software.

### 2.20 VEGFA detection

The cell lysate was prepared and diluted with assay buffer. VEGFA levels were detected by a Human VEGFA ELISA Kit (Invitrogen, USA, #BMS277-2) according to the manufacturer’s instructions.

### 2.21 Flow cytometry analysis

Flow cytometry was performed as previously described (Radomska et al., 2012). Briefly, following treatment, cells were washed once with PBS and blocked in ice-cold PBS containing 2% FBS for 15 min. Cells were then incubated with a FITC-conjugated anti-ITGAX antibody (eBioscience, USA, #11-0116-42) or the corresponding isotype control (Mouse IgG1 kappa Isotype Control, eBioscience, #11-4714-42) at a concentration of 5 µL per 1 × 10^6^ cells (in 100 µL) for 30 min at 4 °C in the dark. After being washed twice with PBS, the cells were resuspended in PBS containing 2% FBS. Fluorescence intensity was acquired using a CytoFLEX flow cytometer (Beckman Coulter, USA), and the mean fluorescence intensity (MFI) was analyzed using CytExpert software.

### 2.22 Co-immunoprecipitation (Co-IP)

For co-immunoprecipitation (Co-IP) of HIF-1α, cells were pre-treated with MG132 (20 μM; MCE, China, #HY13259) for 2 h prior to harvest to prevent proteasomal degradation of HIF-1α. Cells were then lysed in ice-cold IP lysis buffer (MCE, China, #HYK1002) supplemented with a protease inhibitor cocktail (Roche, Switzerland, #04693124001) for 30 min on ice. Lysates were clarified by centrifugation at 12,000 × *g* for 15 min at 4 °C, and protein concentrations were determined by the BCA assay. Equal amounts of total protein (500 μg) were incubated overnight at 4 °C with the primary antibody or isotype control IgG, followed by incubation with protein A/G beads (Thermo Fisher Scientific, USA, #78609) at room temperature for 3 h. The beads were then washed with IP lysis buffer and denatured for Western blot analysis. Antibodies are listed in Table 7.

### 2.23 Prolyl hydroxylase (PHD) activity assay

PHD activity was determined using a PHD activity assay kit (GENMED, China, #GMS50465.1 v.A) based on a coupled NADH oxidation cascade according to the manufacturer’s protocols. Briefly, cells were washed and lysed with dedicated buffers, and the lysates were centrifuged at 16,000 × *g* for 5 min at 4 °C. The supernatant was collected and normalized to a total protein content of 100 μg via a BCA assay (Fdbio Science, China, #FD2001). Concurrently, a reaction mixture containing proline, 2-oxoglutarate, succinyl-CoA synthetase, pyruvate kinase, and lactate dehydrogenase was equilibrated at 37 °C for 3 min. Upon the immediate addition of the sample supernatant or background control, the mixture was subjected to spectrophotometric measurement at 340 nm (37 °C), followed by a second reading after an interval of 30 min. The relative PHD activity (%) was calculated by dividing the absorbance decrease (A_340 nm_) of the sample from 0 to 30 min by that of the background control.

### 2.24 Dual luciferase reporter assay

The human HIF-1α-Flag plasmid was purchased from WZ Biosciences (Shandong, China). For luciferase reporter construction, the ITGAX promoter fragment (−1987 to +1 bp relative to ATG) and its mutant (core motif changed from 5′-AAAGTGCTG-3′ to 5′-GGCACATGT-3′) were synthesized by GENEWIZ (Jiangsu, China) and inserted into the pGL3-basic vector between KpnI and XhoI sites. For reporter assays, HCMECs were transfected with the plasmids using Universal Transfection Reagent (Yeasen, China, #40808ES03). After 48 h, firefly and Renilla luciferase activities were measured using the dual luciferase reporter assay kit (Promega, USA, #E2920).

### 2.25 Tube formation assay

After treatment, HCMECs (7 × 10^4^ per well) were seeded onto Matrigel-coated 24-well plates (Yeasen, China, #40187ES08) in ECM medium for 6 h. Capillary-like structures were imaged using a Nikon inverted phase-contrast microscope and analyzed with NIS-Elements D software (Nikon, Japan).

### 2.26 *In vitro* permeability assay

HCMECs were grown to confluence on 0.4-µm transwell inserts (24-well, Corning, USA, #3470). Then, 100 μL of 1 mg/mL 70 kDa FITC-dextran in ECM medium was added to the upper chamber. After 2 h of free diffusion, fluorescence in the lower chamber was measured using a fluorescence microplate reader (Tecan).

### 2.27 EdU assay

To assess HCMEC proliferation, cells were incubated with 10 μM EdU in ECM medium for 2 h, then fixed with 4% PFA and permeabilized with 0.25% Triton X-100. EdU-positive cells were detected using the BeyoClick™ EdU-594 kit (Beyotime, China, #C0078S) and mounted with fluorescent mounting medium (Beyotime, China, #P0128) for visualization.

### 2.28 Sprouting assay

HCMECs (1.5 × 10^4^ per well) were seeded in U-bottom ultra-low attachment 96-well plates (Beyotime, #FULA962) for 48 h to form 3D spheroids. Spheroids were then transferred to prewarmed ECM medium containing 5 ng/mL vascular endothelial growth factor A (VEGFA; MCE, #HYP700497) and Matrigel (1:1, Yeasen, #40187ES08) in 48-well plates and incubated for 4 h at 37 °C. Sprouting branches were visualized using a Nikon inverted phase-contrast microscope and quantified with ImageJ.

### 2.29 Transwell migration assay

After treatment, HCMECs were seeded in serum-free ECM medium into 8-μm-pore Transwell inserts (24-well, Corning, USA, #3428). Lower chambers contained ECM medium with FBS. After 48 h, cells on the lower membrane surface were stained with 0.2% crystal violet in 10% ethanol (Sangon, China, #E607309) for 15 min, washed three times with water, and imaged using a Nikon ECLIPSE Ni microscope with NIS-Elements D software.

### 2.30 Living cell imaging

HCMECs were transfected with a LifeAct-EGFP lentivirus (obtained from Guannan Biotechnology Company, China) in ECM medium and treated with the appropriate stimuli for 48 h to observe the dynamic reorganization of the actin cytoskeleton. The live-cell images were detected using the LSM900 confocal microscope and the images were analyzed with Zeiss ZEN software.

### 2.31 Quantification and statistical analysis

Data are presented as the mean ± SD and analyzed using GraphPad Prism 9. Normality was assessed by the Shapiro-Wilk test. For normally distributed data: unpaired two-tailed Student’s t-test for two groups; one-way ANOVA followed by Tukey’s test for multiple comparisons; one-way ANOVA followed by Dunnett’s test for comparisons against a control group. Pearson correlation analysis was used for bivariate correlations. All experiments were repeated at least three independent times. A *p value* < 0.05 was considered statistically significant.

## 3 Results

### 3.1 KX attenuates CMVD progression in *db/db* mice

**FIGURE 1:**
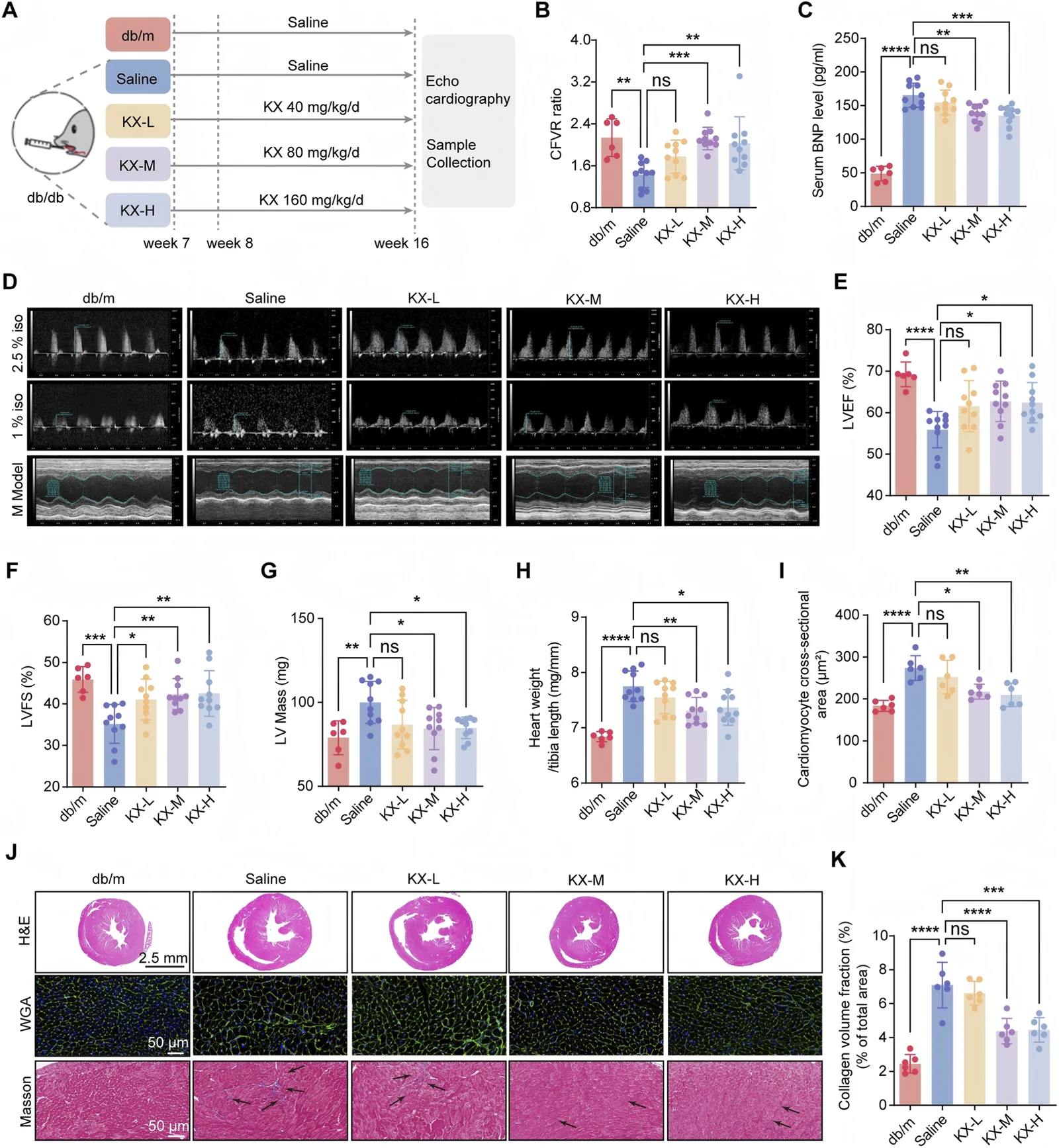
Kuanxiong aerosol ameliorates coronary microvascular dysfunction progression in *db/db* mice. The eight-week-old male *db/db* (*n* = 10) and *db/m* (*n* = 6) mice were sublingually administered Kuanxiong aerosol (KX) (40, 80 and 160 mg/kg, respectively) or saline for 8 weeks. **(A)** Schematic overview of the experimental design. **(B)** Quantitative analysis of coronary flow velocity reserve (CFVR) ratio. **(C)** Serum B-type natriuretic peptide (BNP) levels. **(D–G)** Representative images of CFVR pulse-wave doppler spectrum and mitral inflow assessed by M-mode. Statistical analyses of left ventricular ejection fraction (LVEF), left ventricular fractional shortening (LVFS) and left ventricle mass (LV mass) via transthoracic echocardiography. **(H)** The heart weight to tibia length ratio. **(I–K)** Hematoxylin and eosin (H&E), wheat germ agglutinin (WGA) and Masson’s trichrome staining of heart sections. Representative images **(J)** and quantification of cardiomyocyte cross-sectional area and collagen volume fraction **(I,K)**. Black arrows indicate collagen deposition (*n* = 6). Scale bars: 2.5 mm and 50 µm. Data were presented as mean ± SD. Differences were analyzed by one-way ANOVA with *post hoc* Tukey’s multiple comparisons test. *P < 0.05; **P < 0.01; ***P < 0.001; ****P < 0.0001. ns, no significance.

To evaluate the protective effects of KX against CMVD, *db/db* mice were administered KX daily via sublingual delivery at doses of 40, 80, or 160 mg/kg (low, medium, and high, respectively). The *db/m* mice served as normal controls, while saline-treated *db/db* mice were used as model controls (Figure 1A). Coronary microvascular function was assessed by Doppler echocardiography using the coronary flow velocity reserve (CFVR) ratio. As expected, *db/db* mice exhibited impaired microvascular vasodilatory capacity and increased resistance, as indicated by CFVR values < 2.0. KX treatment dose-dependently improved these abnormalities (Figure 1B). ELISA and echocardiography were performed to assess cardiac function. Compared with controls, *db/db* mice showed significant cardiac dysfunction, characterized by elevated BNP levels and reduced left ventricular ejection fraction (LVEF) and fractional shortening (LVFS). These impairments were partially and dose-dependently ameliorated following KX treatment (Figures 1C–F). Diabetic injury also induced cardiac hypertrophy in *db/db* mice, as evidenced by increased left ventricular mass (LV mass), enlarged cardiac morphology, and increased cardiomyocyte cross-sectional area; these changes were reversed in a dose-dependent manner by KX (Figures 1G–J). Histological analyses further demonstrated pronounced cardiac fibrosis and collagen deposition in *db/db* mice, as revealed by Masson’s trichrome and Picrosirius Red staining. These pathological changes were significantly alleviated after KX treatment (Figures 1J,K; Supplementary Figure S1). Collectively, these findings confirm that KX effectively mitigates CMVD progression in *db/db* mice.

Importantly, prolonged KX administration showed no hepatotoxicity or nephrotoxicity, with key functional markers, including ALT, AST, BUN, CREA and UA remaining unchanged (Supplementary Figure S2). Furthermore, body weight and food intake were comparable between KX-treated and saline-treated *db/db* mice (Supplementary Figures S3A,B). Plasma lipid parameters, including total cholesterol and triglycerides, also showed no significant differences (Supplementary Figures S3C,D). Additionally, KX treatment did not affect glucose metabolism, as indicated by fasting blood glucose levels, oral glucose tolerance tests and insulin tolerance tests (Supplementary Figure S4). Together, these results support the safety profile of KX and suggest that its therapeutic effects on CMVD are independent of alterations in glucose or lipid metabolism.

### 3.2 KX promotes CMVD regression in *db/db* mice

**FIGURE 2:**
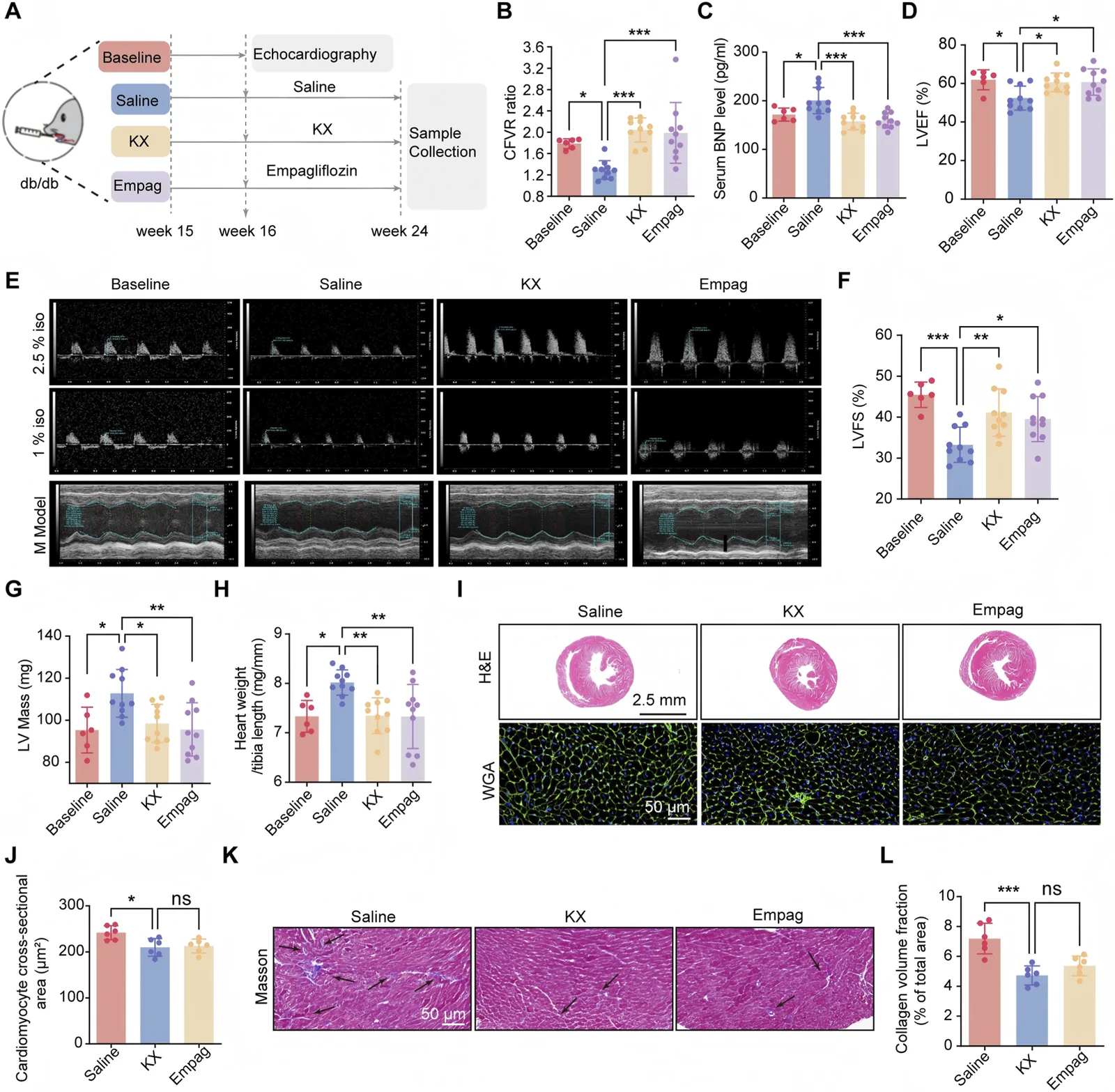
Kuanxiong aerosol promotes coronary microvascular dysfunction regression and cardiac function in *db/db* mice. Sixteen-week-old male *db/db* mice were assigned to a baseline group (euthanized after echocardiography; *n* = 6) or treated for 8 weeks with sublingual Kuanxiong Aerosol (80 mg/kg), intragastric empagliflozin (Empag; 10 mg/kg), or saline vehicle (*n* = 10). **(A)** Schematic overview of the experimental design. **(B)** Quantitative of coronary flow velocity reserve (CFVR) ratio. **(C)** Serum B-type natriuretic peptide (BNP) levels. **(D–G)** Representative images of CFVR pulse-wave doppler spectrum and mitral inflow assessed by M-mode. Statistical analyses of left ventricular ejection fraction (LVEF), left ventricular fractional shortening (LVFS) and left ventricular mass (LV mass) via echocardiography. **(H)** The heart weight to tibia length ratio. **(I,J)** Representative images of Hematoxylin and eosin (H&E) and wheat germ agglutinin (WGA) and quantification of cardiomyocyte cross-sectional area from saline, KX- and empagliflozin-treated *db/db* mice (*n* = 6). Scale bars: 2.5 mm and 50 µm. **(K,L)** Representative images of Masson’s trichrome staining and quantification of collagen volume fraction area. Black arrows represent collagen deposition (*n* = 6). Scale bars: 50 µm. Data as mean ± SD. Differences were analyzed by one-way ANOVA with *post hoc* Tukey’s multiple comparisons test. *P < 0.05; **P < 0.01; ***P < 0.001. ns, no significance.

To examine whether KX could reverse cardiac dysfunction and CMVD in DCM, *db/db* mice were treated daily for 8 weeks with KX (80 mg/kg), empagliflozin (a first-line therapy for CMVD), or saline (Figure 2A). Subsequent CFVR assessment demonstrated that both KX and empagliflozin enhanced microvascular vasodilatory capacity and reduced microvascular resistance (Figure 2B). Consistently, KX or empagliflozin treatment improved cardiac function in *db/db* mice, as evidenced by reduced BNP levels and increased LVEF and LVFS (Figures 2C–F). In addition, KX and empagliflozin reversed cardiac hypertrophy, as indicated by reductions in heart weight, left ventricular mass, overall cardiac enlargement, and cardiomyocyte cross-sectional area (Figures 2G–J). In line with this, Masson’s trichrome and Picrosirius Red staining revealed reduced microvascular stasis and myocardial fibrosis in KX- and empagliflozin-treated mice (Figures 2K,L; Supplementary Figure S5). Thus, these results indicate that KX promotes CMVD regression in *db/db* mice, positioning it as a potent anti-CMVD agent with efficacy comparable to empagliflozin.

### 3.3 KX restores coronary perfusion and maintains endothelial barrier integrity in *db/db* mice

**FIGURE 3:**
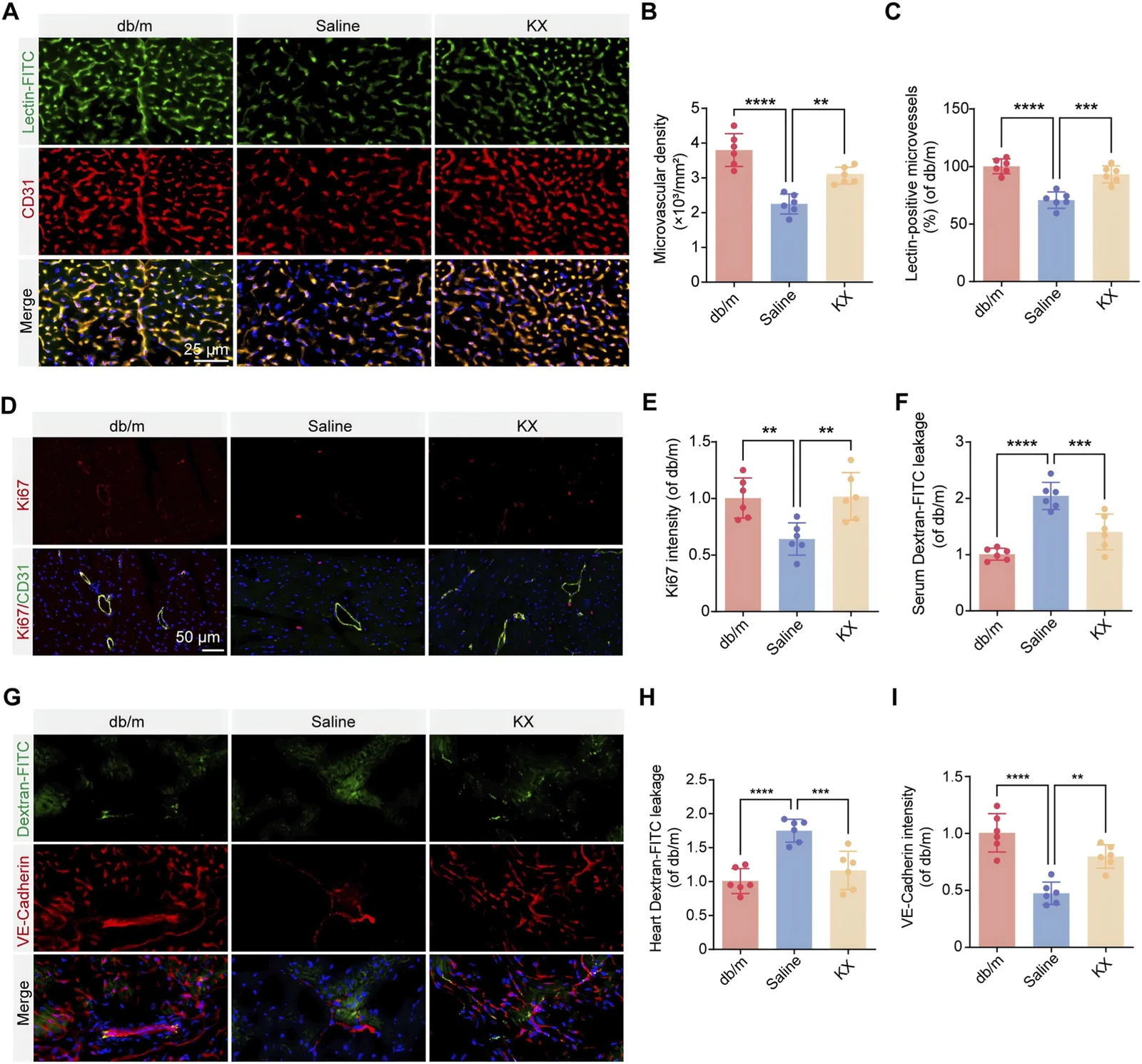
Kuanxiong aerosol restores coronary perfusion and maintains endothelial barrier integrity. The eight-week-old male *db/db* mice were administered Kuanxiong aerosol sublingually at 80 mg/kg or saline once daily for 8 weeks, with age-matched *db/m* mice serving as normal controls (*n* = 6). **(A–C)** Representative images and quantification of CD31-positive microvessels and the normalized cardiac microvascular perfusion, assessed as the ratio of lectin-positive (green) to CD31-positive (red) microvessels. Scale bars: 25 µm. **(D,E)** Analysis of myocardial endothelial proliferation in *db/db* and *db/m* mice. Representative immunofluorescence images and normalized quantification of Ki67-positive areas (red). Scale bars: 50 µm. **(F–I)** Analysis of myocardial endothelial barrier permeability in *db/db* and *db/m* mice. Serum dextran-FITC leakage level was detected by *in vivo* vascular permeability assay **(F)**. **(G–I)** Representative images and the quantification of dextran-FITC leakage and the normalized VE-Cadherin -positive areas (red). Scale bars: 25 µm. All values were normalized to the *db/m* group. Data were presented as mean ± SD. Differences were analyzed by one-way ANOVA with *post hoc* Tukey’s multiple comparisons test. **P < 0.01; ***P < 0.001; ****P < 0.0001.

Obesity- and diabetes-associated coronary microvascular dysfunction is pathologically characterized by endothelial dysfunction, capillary rarefaction and increased vascular permeability, which collectively compromise coronary perfusion and drive myocardial remodeling toward heart failure (Qiu et al., 2022). To investigate whether KX attenuates CMVD progression by preserving coronary microvascular perfusion and barrier integrity, lectin-based coronary perfusion, cardiac microvascular density and Dextran-FITC leakage assays were performed *in vivo*. KX treatment significantly ameliorated microvascular density and perfusion capacity in *db/db* mice (Figures 3A–C). Furthermore, immunofluorescence staining revealed that KX treatment markedly rescued the proliferative capacity of coronary microvascular endothelial cells, which was substantially diminished under chronic diabetic conditions (Figures 3D,E). Regarding vascular barrier integrity, prolonged diabetic injury resulted in microvascular hyperpermeability and disruption of anchoring junctions, as evidenced by increased Dextran-FITC leakage and reduced VE-cadherin expression; KX treatment effectively reversed these pathological alterations (Figures 3F–I). Collectively, these findings demonstrate that KX protects against CMVD by restoring coronary microvascular perfusion and reinforcing endothelial barrier integrity.

### 3.4 KX improves angiogenesis and preserves neovascular integrity in HGHF-injured HCMECs

**FIGURE 4:**
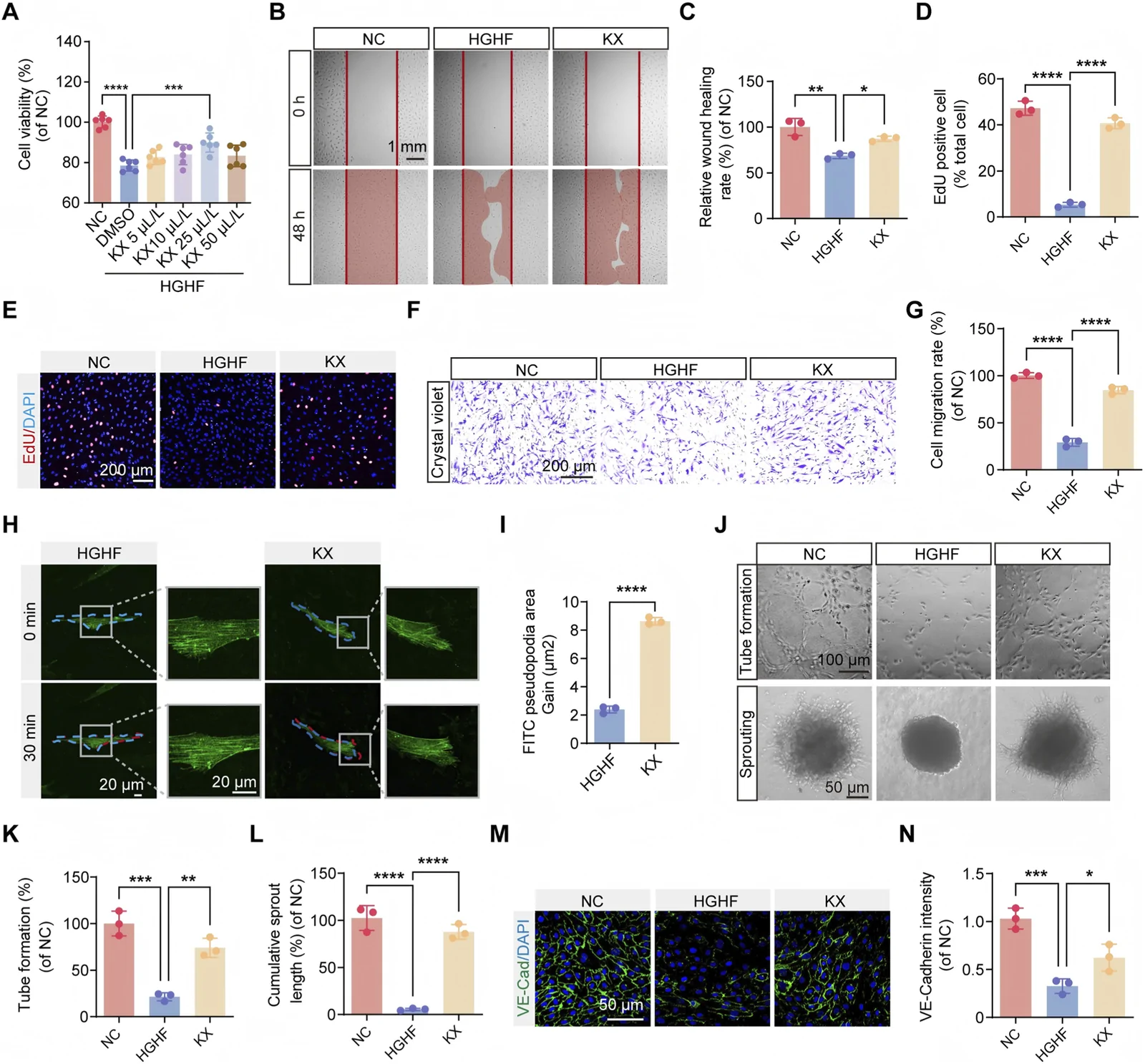
Kuanxiong aerosol enhances angiogenesis and preserves neovascular integrity in HGHF-injured HCMECs. **(A)** Cell Counting Kit-8 (CCK-8) assay for cell viability. Human cardiac microvascular endothelial cells (HCMECs) were treated with 5, 10, 25, 50 μL/L Kuanxiong aerosol (KX) and DMSO vehicle for 48 h under high glucose and high fat (HGHF) injury (*n* = 6). **(B,C)** HCMECs were treated with KX or DMSO under HGHF condition or not (*n* = 3). Representative images and normalized quantification of wound healing area. Scale bar, 1 mm. **(D,E)** Representative EdU staining images and normalized proliferation rates in HCMECs described in **(B)**. Scale bar, 200 μm. **(F,G)** Representative images and normalized migration rate of HCMECs described in **(B)**. Scale bar, 200 μm. **(H,I)** Representative living-cell motility images and quantification of pseudopod area in HGHF-injured HCMECs with KX or DMSO treatment (*n* = 3). **(J–L)** Representative tube formation and sprouting images and the normalized quantification of tube formation rate and sprouting length of HCMECs described in **(B)**. Scale bar, 100 μm and 50 μm. **(M,N)** Representative immunofluorescence images and normalized quantification VE-Cadherin in HCMECs described in **(B)**. Scale bar, 50 μm. Data are presented as mean ± SD. Differences were analyzed by one-way ANOVA with *post hoc* Tukey’s multiple comparisons test **(A,C,D,G,K,L,N)** and unpaired t-test **(I)**. *P < 0.05; **P < 0.01; ***P < 0.001; ****P < 0.0001.

To further elucidate the effects of KX on microvascular angiogenesis and endothelial barrier integrity under HGHF injury, a series of functional assays were performed in HCMECs. CCK-8 assays identified 25 μL/L as the optimal KX concentration for promoting cell viability. LDH release assays confirmed the absence of non-specific cytotoxicity at this concentration; notably, HGHF-injured cells exhibited marked LDH elevation indicative of membrane rupture, whereas KX treatment attenuated this pathological release, demonstrating endothelial cytoprotective efficacy (Figure 4A; Supplementary Figure S6). Angiogenesis is a tightly regulated process that depends on coordinated endothelial cell proliferation, migration and tubulogenesis (Herbert and Stainier, 2011). To assess the pro-angiogenic effects of KX, HGHF-injured HCMECs were treated with KX or vehicle control. Specifically, KX treatment significantly enhanced endothelial cell proliferation, as evidenced by wound healing and EdU incorporation assays (Figures 4B–E). Furthermore, KX markedly promoted cell migration under HGHF conditions, as demonstrated by Transwell assays (Figures 4F,G) and live-cell imaging of pseudopodial area (Figures 4H,I). Consistent with these findings, KX also enhanced endothelial sprouting and tube formation capacity (Figures 4J–L). During microvascular angiogenesis, VE-cadherin stabilizes endothelial adherens junctions and cooperates with integrins to support nascent lumen formation (Nan et al., 2023) (Vestweber, 2008). VE-cadherin expression and junctional organization were markedly enhanced by KX treatment, as shown by immunofluorescence staining (Figures 4M,N). These findings indicate that KX promotes angiogenesis and preserves neovascular integrity in HGHF-injured HCMECs.

### 3.5 ITGAX upregulation mediates the pro-angiogenic and endoprotective effects of KX

**FIGURE 5:**
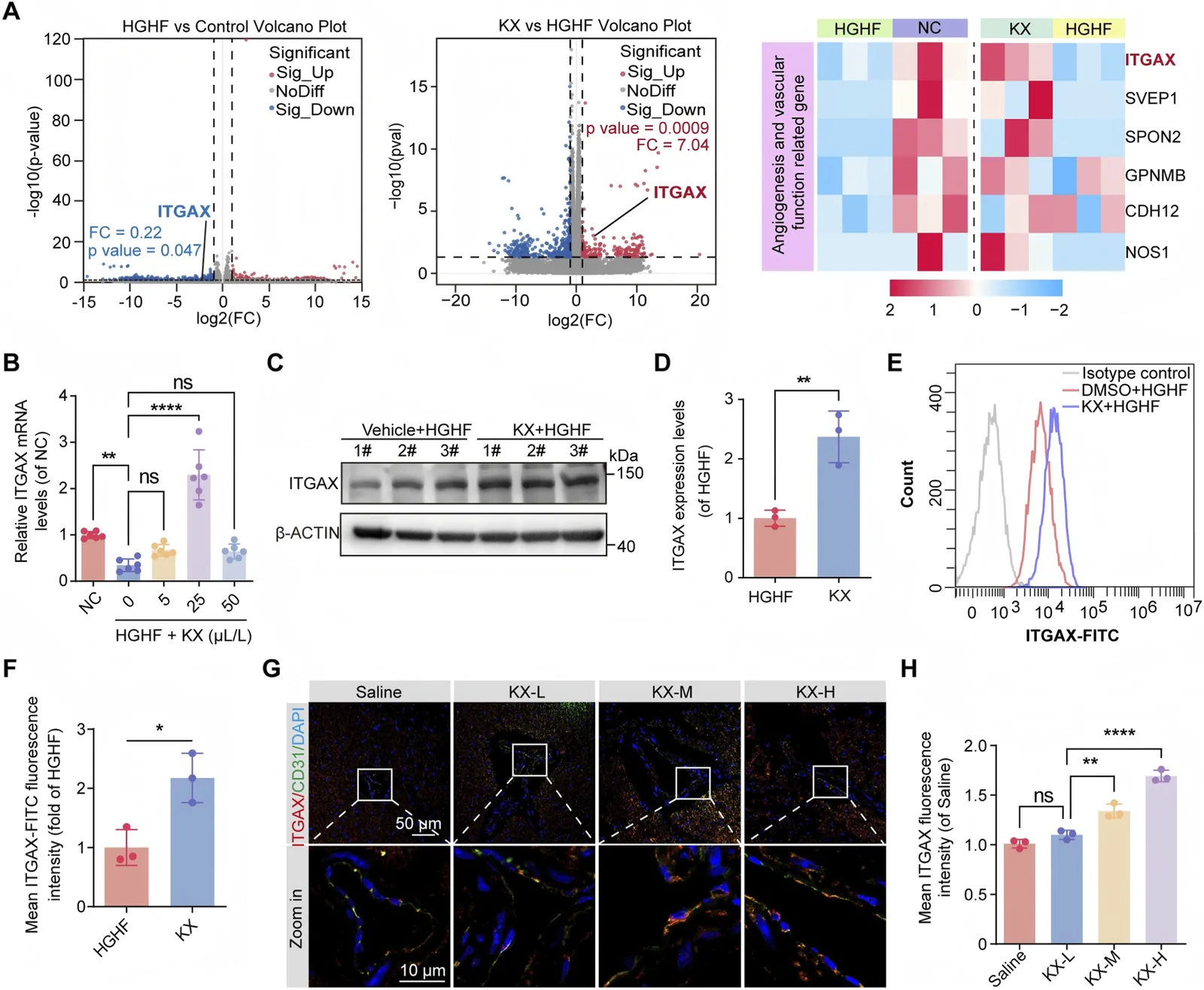
ITGAX upregulation mediates the pro-angiogenic and endoprotective effects of Kuanxiong aerosol **(A)** Volcano plot displaying differentially expressed genes (DEGs) from bulk RNA-seq analysis of HCMECs under 48 h DMSO or high glucose and high fatty acid (HGHF) conditions, with or without 25 μL/L Kuanxiong aerosol (KX). The heatmap of DEGs related to angiogenesis and endothelial function. **(B)** Normalized ITGAX mRNA levels in HCMECs treated with KX or DMSO under HGHF condition or not (*n* = 6). **(C,D)** Representative immunoblots and normalized quantification of ITGAX in HGHF-injured HCMECs with KX or DMSO treatment. **(E,F)** Representative flow cytometry plots and normalized mean fluorescence intensity of ITGAX-FITC in HGHF-injured HCMECs with KX or DMSO treatment. **(G,H)** Representative immunofluorescence images and normalized quantification of ITGAX from *db/db* mice with 8-week 40, 80 and 160 mg/kg KX or saline treatment (*n* = 6). Scale bar, 50 and 10 μm. Data were presented as mean ± SD. Differences were analyzed by one-way ANOVA with *post hoc* Tukey’s multiple comparisons test **(B,H)** and unpaired t-test **(D,F)**. **P < 0.01; ****P < 0.0001. ns, no significance.

To pinpoint the core molecular targets underlying the protective effects of KX against CMVD, bulk RNA sequencing was performed comparing HGHF-injured with normal HCMECs, as well as KX-treated with vehicle-treated HGHF-injured HCMECs. Cross-comparison of the resulting differentially expressed genes revealed a cluster of candidates closely implicated in angiogenesis and endothelial function. Among these, Integrin Subunit Alpha X (ITGAX) was significantly downregulated (0.22-fold) under HGHF injury and markedly restored (7-fold) following KX treatment (Figure 5A). As a protein known to promote endothelial angiogenesis and nascent vessel stabilization under hypoxia via integrin-mediated tight junctions (Davis and Senger, 2005; Wang et al., 2019), ITGAX likely represents a target of KX during CMVD progression. Subsequent biochemical and flow cytometry analyses confirmed that KX treatment significantly upregulated ITGAX in HGHF-injured HCMECs at the mRNA and protein levels (Figures 5B–F). Consistently, immunofluorescence staining revealed a dose-dependent increase of ITGAX in the coronary microvasculature of KX-treated *db/db* mice (Figures 5G,H). Collectively, these findings demonstrated that upregulation of ITGAX constitutes a key molecular link between KX treatment and enhanced angiogenesis with preserved neovascular integrity in CMVD.

### 3.6 KX upregulates ITGAX expression by stabilizing HIF-1α against PHD2-mediated proteasomal degradation

**FIGURE 6:**
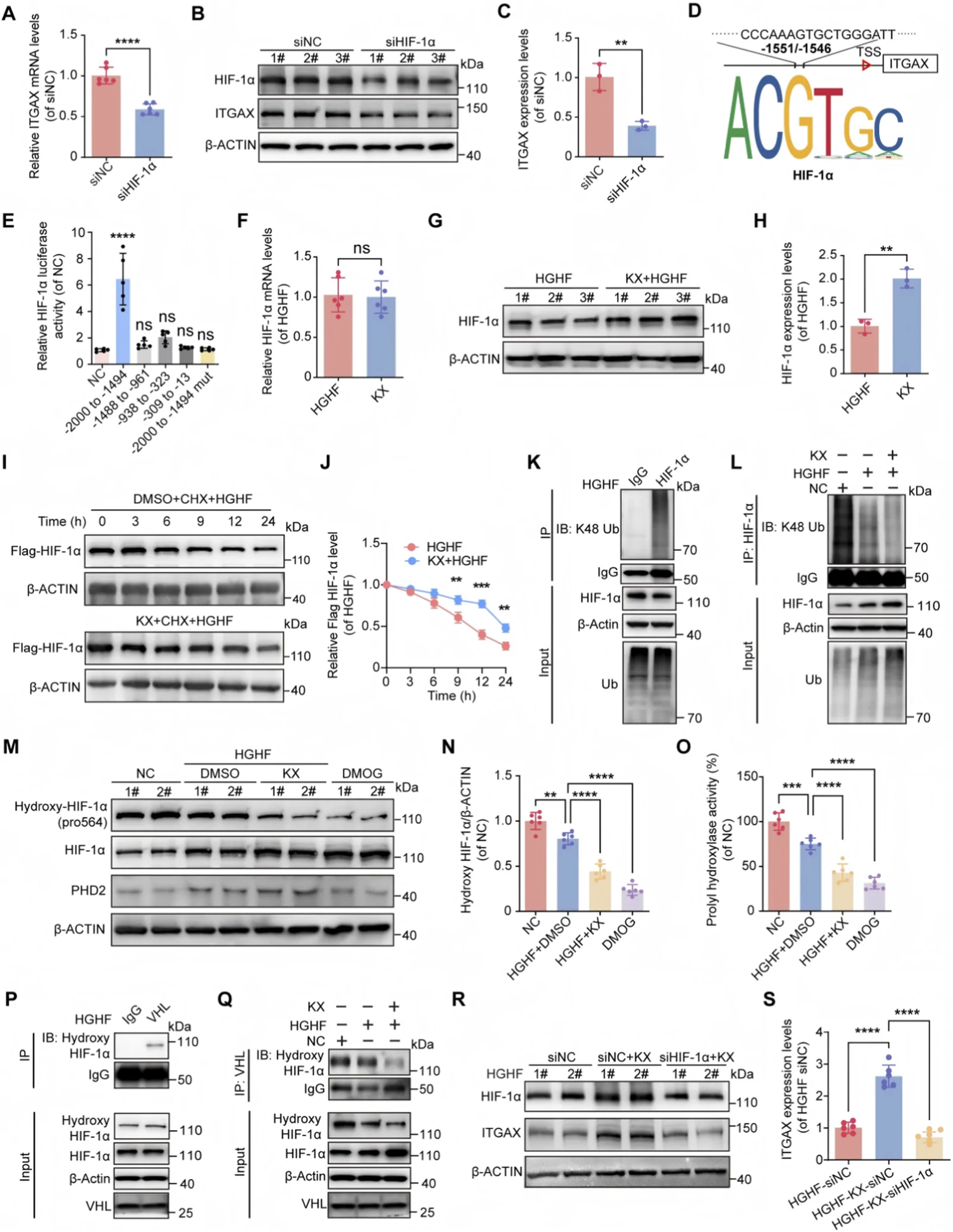
Kuanxiong aerosol elevates ITGAX expression via stabilizing its transcription factor HIF-1α. **(A–C)** Normalized ITGAX mRNA in siNC or siHIF-1α HCMECs (*n* = 6). Representative blots and normalized quantification of ITGAX and HIF-1α (*n* = 3). **(D)** ITGAX promoter binding motifs predicted by database analysis. **(E)** Luciferase activity in HCMECs 48 h post-transfection with ITGAX promoter segments and HIF-1α, normalized to Renilla and negative controls (*n* = 5). **(F–H)** HCMECs were treated with 25 μL/L Kuanxiong aerosol (KX) or DMSO for 48 h under the high glucose and high fatty acid (HGHF) condition (*n* = 6). Representative blots and normalized HIF-1α mRNA and protein levels. **(I,J)** HIF-1α protein stability in Flag-HIF-1α-transfected HCMECs treated with KX or DMSO under HGHF conditions with 100 μg/mL cycloheximide (CHX) for the indicated times. Representative blots and normalized quantification of HIF-1α (*n* = 3). **(K,L)** Co-Immunoprecipitation (Co-IP) detection of HIF-1α ubiquitination by K48-linked ubiquitin (K48-Ub) in HCMECs subjected to HGHF injury **(K)** or treated with KX under normal or HGHF conditions **(L)**. **(M–O)** HCMECs under normal or HGHF conditions were treated with KX, DMSO, or the prolyl hydroxylase (PHD) inhibitor dimethyloxalylglycine (DMOG; 1 mM) for 6 h (*n* = 6). Representative blots and normalized quantification of HIF-1α, hydroxy-HIF-1α and PHD2 **(M,N)**. The normalized PHD enzyme activity **(O)**. **(P,Q)** Co-IP analysis of interaction between hydroxy-HIF-1α and VHL in HGHF-injured HCMECs **(P)** or treated with KX under normal or HGHF conditions **(Q)**. **(R,S)** Representative blots and normalized quantification of ITGAX and HIF-1α in siNC or siHIF-1α HCMECs with KX or DMSO under HGHF condition (*n* = 6). Data as mean ± SD. Differences were analyzed by unpaired t-test **(A,C,F,H)**, two-way repeated measures ANOVA with Bonferroni multiple comparisons test **(J)** and one-way ANOVA with *post hoc* Tukey’s multiple comparisons test **(E,N,O,S)**. **P < 0.01; ***P < 0.001; ****P < 0.0001. ns, no significance.

To elucidate the regulatory mechanism governing KX-induced ITGAX expression, we systematically interrogated bioinformatic databases for potential upstream transcription factors. This bioinformatic analysis revealed a potential regulatory link between ITGAX and HIF-1α and a putative HIF-1α binding motif within the ITGAX promoter region (Supplementary Figure S7). Subsequent multiple-sequence alignment further demonstrated that this binding site is highly conserved across diverse species, suggesting a critical and evolutionarily conserved regulatory link between HIF-1α and ITGAX. Consistently, siRNA-mediated knockdown of HIF-1α significantly suppressed both the mRNA and protein expressions of ITGAX in HCMECs (Figures 6A–C). Dual-luciferase reporter assays further confirmed that HIF-1α drives ITGAX transcription through a specific promoter region located between −1551 and −1546 bp. Notably, site-directed mutagenesis of this specific motif completely abrogated the luciferase activity, substantiating its identity as a functional, non-canonical hypoxia response element (HRE) (Figures 6D,E). Collectively, these findings establish HIF-1α as a novel upstream transcription factor that directly transactivates ITGAX.

Importantly, biochemical assays showed that while HIF1A mRNA levels in HGHF-injured HCMECs remained unaffected by KX treatment (Figure 6F), its protein expression was significantly increased (Figures 6G,H). Additionally, a cycloheximide (CHX) chase assay indicated that KX markedly enhanced HIF-1α protein stability (Figures 6I,J), suggesting that KX elevates HIF-1α transcriptional activity via post-translational stabilization rather than transcriptional upregulation. In endothelial cells, dominant PHD2 controls HIF-1α levels by prolyl hydroxylation, directing HIF-1α to VHL-mediated proteasomal degradation (Huang et al., 2002). Co-immunoprecipitation (Co-IP) assays revealed that KX effectively suppressed the proteasome-dependent ubiquitination of HIF-1α, characterized by a substantial reduction in K48-linked polyubiquitination (Figures 6K,L).

To delineate whether this stabilization stems from the impairment of PHD2-mediated hydroxylation, we evaluated PHD2 activity and hydroxy-HIF-1α levels under glucolipotoxic stress, using DMOG as a positive control. KX treatment significantly blunted PHD2 enzymatic activity and suppressed hydroxy-HIF-1α accumulation (Figures 6M–O). Given that the E3 ligase VHL selectively recognizes hydroxy-HIF-1α to catalyze ubiquitination (Huang et al., 2002), we performed Co-IP to assess their physical association. Consistently, the KX-induced reduction in hydroxy-HIF-1α secondary to PHD2 inhibition significantly disrupted VHL recruitment to hydroxy-HIF-1α (Figures 6P,Q). Furthermore, to verify whether the upregulation of ITGAX by KX requires HIF-1α, we silenced HIF-1α prior to KX treatment. It is found that this genetic deficiency entirely abolished the capability of KX to induce ITGAX expression (Figures 6R,S). Consequently, KX stabilizes HIF-1α against PHD2-mediated proteasomal degradation, thereby driving the adaptive upregulation of ITGAX in HGHF-injured HCMECs.

### 3.7 HIF-1α knockout abrogates the protective effects of KX against HCMECs dysfunction

**FIGURE 7:**
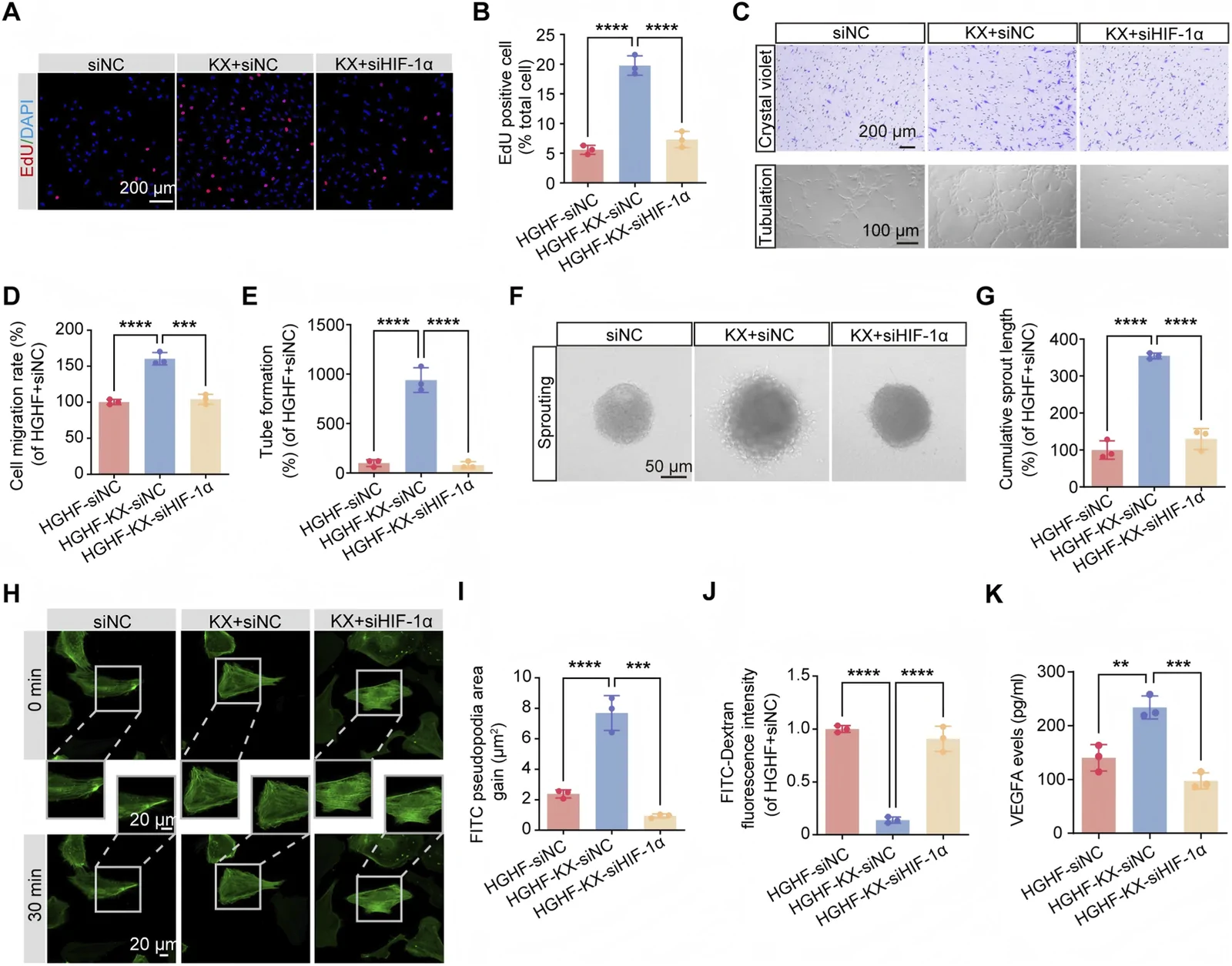
HIF-1α knockout abrogates the protective effects of Kuanxiong aerosol against HCMEC dysfunction. The HCMECs were transfected with siNC or siHIF-1α for 24 h, followed by treatment with 25 μL/L KX or DMSO under high glucose and high fatty acid (HGHF) treatment for another 48 h before harvesting. (*n* = 3) **(A,B)** Representative EdU staining image and normalized quantification of HCMECs proliferation. Scale bar, 200 μm. **(C–E)** Representative crystal violet staining and tube formation images, and normalized quantification of migration and tube formation rates in HCMECs. Scale bar, 100 μm and 200 μm, respectively. **(F,G)** Representative sprouting images and normalized quantification of sprouting length in HCMECs. Scale bar, 50 μm. **(H,I)** Representative living-cell images of cell motility and normalized quantification of pseudopod area in HCMECs. Scale bar, 20 μm. **(J)** The *in vitro* permeability assay for detecting HCMECs endothelial barrier integrity. **(K)** The VEGFA levels of HCMECs. Values were normalized to the HGHF + siNC group. Data were presented as mean ± SD. Differences were analyzed by one-way ANOVA with *post hoc* Tukey’s multiple comparisons test. **P < 0.01; ***P < 0.001; ****P < 0.0001.

To elucidate whether KX exerts its protective effects against HGHF-induced endothelial dysfunction through HIF-1α, a loss-of-function approach was employed by transfecting HCMECs with HIF-1α-targeting siRNA prior to KX or vehicle administration. Under HGHF conditions, HCMECs exhibited a profound impairment in their angiogenic and barrier capacities, which was significantly rescued by KX treatment. However, this KX-mediated therapeutic efficacy was largely neutralized by HIF-1α silencing. Specifically, HIF-1α knockdown significantly reversed the KX-enhanced cell proliferation (Figures 7A,B) and compromised its promotive effects on cell migration (Figures 7C,D). Furthermore, the substantial improvements in angiogenic potential following KX treatment, which were evidenced by enhanced tube formation, *ex vivo* sprouting (Figures 7E–G), and pseudopodia area recovery (Figures 7H,I), were effectively abolished in the absence of HIF-1α. In parallel, FITC-dextran permeability revealed that HIF-1α deficiency also negated the protective role of KX in preserving endothelial barrier integrity under glucolipotoxic stress (Figure 7J). Notably, KX enhanced the secretion of VEGFA in HGHF-injured HCMECs, an effect abolished by HIF-1α knockdown (Figure 7K), consistent with the known role of HIF-1α in transactivating VEGFA via hypoxia response elements (Jiang et al., 2020; Wen et al., 2019), suggesting that KX triggers an adaptive angiogenesis mechanism to counteract metabolic injury. Consequently, these phenotypic reversals underscore that HIF-1α acts as a critical molecular required for KX to drive adaptive angiogenesis and sustain endothelial homeostasis under metabolic stress.

### 3.8 KX accelerates angiogenesis and sustains microvascular integrity by activating the HIF-1α-ITGAX axis and VEGF-FAK signaling

**FIGURE 8:**
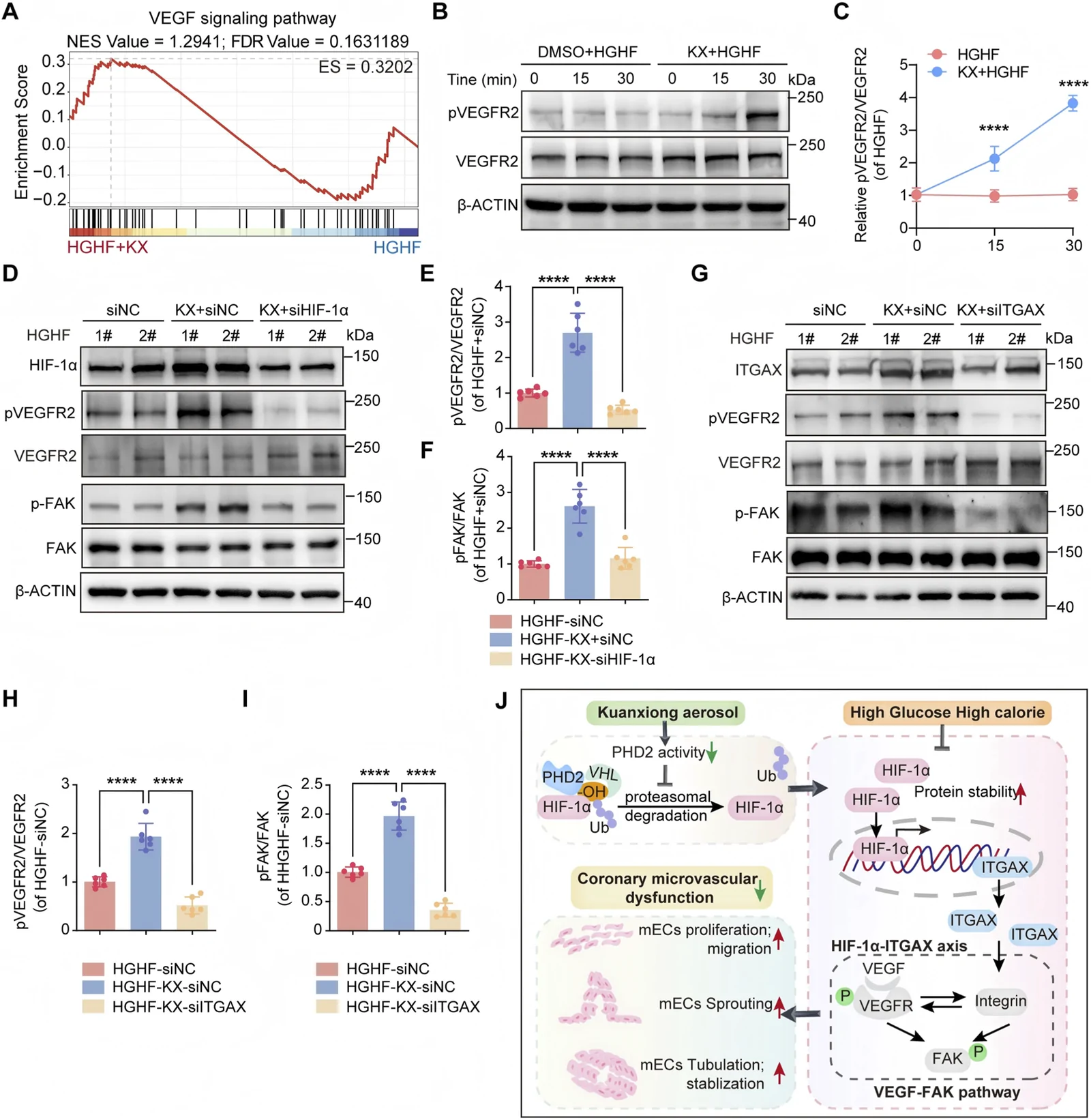
Kuanxiong aerosol accelerates angiogenesis and sustains microvascular integrity by activating the HIF-1α-ITGAX axis and VEGF-FAK signaling. **(A)** Gene set enrichment analysis showing enrichment of the VEGF signaling pathway in high glucose and high fatty acid (HGHF)-injured HCMECs treated with Kuanxiong aerosol (KX) versus DMSO vehicle. **(B,C)** HCMECs were treated with DMSO or 25 μL/L KX for 0, 15, and 30 min under HGHF condition. Representative blots and normalized quantification of pVEGFR2 and VEGFR2 (*n* = 3). **(D–F)** HCMECs were transfected with siNC or siHIF-1α for 24 h, followed by treatment with KX or DMSO for another 48 h under HGHF condition (*n* = 6). Representative immunoblots and quantification of HIF-1α, pVEGFR2/VEGFR2 and pFAK/FAK in HCMECs. **(G–I)** HCMECs were transfected with siNC or siITGAX for 24 h, followed by treatment with KX or DMSO vehicle under HGHF treatment for another 48 h (*n* = 6). Representative immunoblots and quantification of ITGAX, pVEGFR2, VEGFR2, pFAK and FAK in HCMECs. Values were normalized to the HGHF + siNC group. Data are presented as the mean ± SD. Differences were analyzed by one-way ANOVA with post hoc Tukey’s multiple comparisons test **(E,F,H,I)** and two-way ANOVA with Bonferroni multiple comparisons test **(C)**. ****P < 0.0001. **(J)** Mechanistic model of the protective effects of KX against coronary microvascular dysfunction (CMVD) in diabetes mellitus.

To elucidate the specific pathway through which KX accelerates angiogenesis and sustains microvascular integrity, we performed Gene Set Enrichment Analysis (GSEA) on RNA-sequencing data. Notably, transcriptomic profiling revealed that KX treatment was positively correlated with the activation of the VEGF signaling pathway in HGHF-injured HCMECs (Figure 8A). Concurring with the VEGFR2 signaling cascade is well-documented as the indispensable gatekeeper of endothelial cell proliferation and adaptive angiogenesis (Wang et al., 2019; Huang et al., 2002), Western blot analysis validated that KX significantly elevated the phosphorylation levels of VEGFR2 (Figures 8B,C). Intriguingly, this activation was effectively abolished by HIF-1α knockdown (Supplementary Figure S8), establishing that KX acts upstream to fortify the VEGF cascade in a HIF-1α-dependent manner under glucolipotoxic conditions. Moreover, Focal adhesion kinase (FAK) signaling serves as a vital molecular node linking integrin engagement and VEGF stimulation to the maintenance of endothelial homeostasis (Chen et al., 2012; Li et al., 2025). It is found that KX-induced activation of the VEGF-FAK pathway was substantially diminished in both HIF-1α-deficient HCMECs (Figures 8D–F) and ITGAX-knockdown cells (Figures 8G–I), suggesting that the HIF-1α-ITGAX axis may play a key role in mediating the protective efficacy of KX against HGHF-induced endothelial injury.

In summary, KX orchestrates the HIF-1α-ITGAX axis to drive integrin-dependent adaptive angiogenesis and barrier preservation via the downstream VEGF-FAK signaling pathway, thereby safeguarding HCMECs against glucolipotoxic stress and highlighting its therapeutic promise for coronary microvascular dysfunction (Figure 8J).

## 4 Discussion

CMVD is increasingly recognized as a key driver of heart failure in the setting of diabetes, yet effective therapies that directly target the coronary microcirculation remain limited. In this study, we demonstrated that KX protects against CMVD by engaging a concise endothelial signaling cascade centered on the HIF-1α-ITGAX axis and the VEGF-FAK pathway, thereby enhancing angiogenesis and restoring myocardial microvascular perfusion.

The role of hypoxia-inducible factor signaling in diabetic cardiovascular complications remains controversial; chronic hyperglycemia typically impairs HIF-1α stabilization, compromising adaptive angiogenesis, whereas excessive activation can drive pathological neovascularization and inflammation (Catrina et al., 2004; Thangarajah et al., 2009). Our findings further elucidate this regulatory nuance by demonstrating that under metabolic stress, KX post-translationally stabilizes HIF-1α to restore physiological angiogenic signaling without inducing uncontrolled vessel sprouting. Mechanistically, PHD2 is a 2-oxoglutarate-dependent dioxygenase that senses oxygen to control HIF-1α stability via prolyl hydroxylation (Kaelin and Ratcliffe, 2008). Beyond its canonical dependence on oxygen, ferrous iron, and 2-oxoglutarate (Kaelin and Ratcliffe, 2008; Berra et al., 2003), PHD2 activity is further fine-tuned by cellular redox dynamics (Lee et al., 2016) and diverse post-translational modifications (Di et al., 2017). In endothelial cells, PHD2 acts as the predominant gatekeeper of HIF-1α stability, directing its VHL-mediated proteasomal degradation (Jaakkola et al., 2001; Ivan et al., 2001); indeed, accumulating evidence suggests that endothelial PHD2 is an important determinant of vascular barrier integrity and homeostasis (Fan et al., 2019; Takeda and Fong, 2007; Ohh et al., 2000). Consistently, our biochemical analyses reveal that KX interrupts this canonical cascade by blunting PHD2 enzymatic activity and reducing hydroxy-HIF-1α accumulation, thereby impairing E3 ligase VHL recruitment and subsequent K48-linked polyubiquitination. Although our data show that KX suppresses PHD2 activity, whether this occurs via direct binding or indirect modulation remains to be elucidated and will require future biochemical validation and targeted redox interrogation. Crucially, silencing HIF-1α markedly attenuated the ability of KX to induce downstream ITGAX expression, supporting the functional relevance of this stabilized axis. By fine-tuning the PHD2-VHL-HIF-1α pathway to address microvascular rarefaction and perfusion deficits while limiting aberrant sprouting, KX appears to align with an emerging paradigm in anti-diabetic cardiovascular therapy that favors restoration of physiological hypoxia responsiveness over global amplification of systemic hypoxic signaling (Bento and Pereira, 2011; Fagundes et al., 2024; Fang et al., 2025).

HIF-1α is a master regulator of vascular homeostasis, controlling key angiogenic mediators such as VEGF (Catrina and Zheng, 2021). Under diabetic conditions, its stability and transcriptional activity are frequently impaired, compromising adaptive angiogenesis and increasing susceptibility to cardiovascular complications (Sato and Takeda, 2023; Thangarajah et al., 2010; Ullah and Wu, 2021; Li et al., 2021). While effective microvascular repair requires endothelial sprouting, durable and perfusion-competent neovessels ultimately depend on subsequent stabilization and functional integration mediated by integrin-extracellular matrix interactions (Davis and Senger, 2005; Hynes, 2007). Beyond its classical role in immune cell adhesion, ITGAX has recently emerged as a novel regulator of hypoxia-induced angiogenesis (Wang et al., 2019; Befani and Liakos, 2017). Here, we establish ITGAX as a direct transcriptional target of HIF-1α in endothelial cells, linking hypoxic adaptation to integrin-dependent vascular stabilization. Nevertheless, although our data underscore ITGAX’s functional contribution, whether its induction is independently sufficient to drive the full spectrum of KX-mediated endothelial protection and angiogenesis both *in vitro* and *in vivo* remains to be fully determined. Given that HIF-1α orchestrates complex gene regulatory networks, it represents the primary determinant of these phenotypes, whereas ITGAX likely acts as a specialized downstream effector cooperating with other canonical targets to fine-tune vascular remodeling.

Our dual-luciferase reporter assays demonstrate that HIF-1α directly transactivates ITGAX via a functional, non-canonical hypoxia response element (HRE). Although the 5′-RCGTG-3′ motif is the widely recognized canonical binding site, accumulating evidence confirms that HIF-1α can transactivate target cardiovascular genes via atypical elements, such as guanine-rich promoter regions in Notch3 regulation (Nakamura et al., 2022) or non-canonical sequences within the VLDLr promoter (Sundelin et al., 2013). Crucially, site-directed mutation of this newly identified core sequence markedly reduced KX-induced ITGAX promoter activity, providing direct functional evidence that such non-canonical HRE motifs constitute critical regulatory elements governing endothelial function and vascular integrity under metabolic stress. Building on this transcriptional foundation, KX acts upstream to modulate the VEGF cascade, orchestrating a critical molecular crosstalk. Given that FAK signaling is a critical hub connecting integrin engagement and VEGF stimulation to endothelial homeostasis (Chen et al., 2012; Li et al., 2025), our data indicate that KX acts at this nexus to reinforce endothelial stability and angiogenic capacity. Remarkably, KX-induced activation of the VEGF/FAK pathway was substantially diminished not only in HIF-1α-deficient cells but also in ITGAX-knockdown cells, indicating that the protective efficacy of KX against glucolipotoxic injury is largely dependent on the HIF-1α-ITGAX axis. Through this intricate signaling cascade, KX functionally enhanced VE-cadherin integrity, reduced dextran leakage, and improved coronary flow velocity reserve, thereby pointing to a simultaneous restoration of endothelial barrier function and perfusion capacity. However, siRNA-mediated silencing of HIF-1α and ITGAX achieved only partial protein-level knockdown, which may underlie residual VEGF-FAK activity and incomplete attenuation of KX-mediated endothelial protection. Thus, these residual effects are more likely due to incomplete target suppression rather than compensatory or HIF-1α/ITGAX-independent mechanisms. Future studies using CRISPR-Cas9 or inducible knockdown systems may further define the contribution of the HIF-1α-ITGAX axis to KX’s protective effects. Collectively, these findings suggest that a HIF-1α-driven, ITGAX-dependent signaling axis contributes to the protective effects of KX on endothelial function and vascular homeostasis under glucolipotoxic stress.

Pharmacokinetically, KX is delivered via a sublingual spray, allowing direct mucosal absorption into the systemic circulation and avoiding hepatic first-pass metabolism. The formulation predominantly comprises volatile, lipophilic constituents with physicochemical properties favorable for transmucosal absorption. Although comprehensive pharmacokinetic characterization of the full formulation remains limited, rat *in vivo* studies have demonstrated rapid systemic absorption of 1,8-cineole, a representative KX constituent, with a time to peak plasma concentration (T_max_) of 0.05 h following sublingual administration (Wu et al., 2020). Clinically, multicenter randomized controlled studies (RCTs) consistently reported 3- and 5-min angina relief rates comparable to or exceeding those of nitroglycerin (Yang et al., 2018; Huang et al., 2024), indirectly supporting prompt systemic exposure. Regarding safety, a meta-analysis of 12 RCTs (n = 2,001) reported a significantly lower incidence of adverse reactions with KX compared with nitroglycerin (RR = 0.42, 95% CI: 0.33–0.54), with no clinically significant hepatic or renal abnormalities over 8 weeks (Zhuang et al., 2020), findings that were further corroborated by a multicenter RCT (n = 194) over 12 weeks (Huang et al., 2024). Collectively, current evidence indicates a favorable safety profile for KX; however, systematic pharmacokinetic studies of the intact formulation remain warranted.

Characterized by its multi-component formulation, KX inherently exerts a multifaceted, integrative regulatory network across diverse vascular cell lineages. While Chen et al. reported that KX suppresses plaque inflammation via the macrophage CX3CR1 pathway (Chen et al., 2025), our findings reveal a distinct, direct mechanism wherein KX activates the endothelial HIF-1α-ITGAX axis. Operating independently across distinct cell types, these parallel arms functionally converge to maintain vascular wall homeostasis, surpassing the conventional single-target monotherapy paradigm. Fundamentally, KX delivers systemic cardiovascular efficacy through a multi-track strategy: depressing extrinsic, myeloid-driven inflammation via macrophage CX3CR1 signaling, while concurrently preserving intrinsic endothelial homeostasis, angiogenesis, and barrier integrity through the HIF-1α-ITGAX pathway to combat coronary microvascular dysfunction. This cell-type-specific synergy underscores the holistic therapeutic advantage of KX in managing multifaceted cardiovascular disorders.

Furthermore, the comparison with empagliflozin further strengthens the translational relevance of our findings. Sodium-glucose cotransporter 2 (SGLT2) inhibitors, including empagliflozin, have demonstrated robust cardiovascular benefits in patients with DCM, mediated in part by improvements in microvascular function, myocardial energetics, and anti-inflammatory effects (Packer et al., 2020; Anker et al., 2021). Notably, in our study, KX improved CMVD without altering glucose metabolism, lipid profiles, or body weight, indicating a mechanism that is largely independent of systemic metabolic correction. This feature is clinically relevant, as a substantial proportion of CMVD patients present with normoglycemia or well-controlled diabetes but continue to exhibit microvascular dysfunction. Thus, KX may serve as a complementary or adjunctive strategy to directly promote endothelial repair. Taken together, our findings provide mechanistic insight and a translational perspective, supporting KX as a candidate therapy that targets the endothelial angiogenic niche in CMVD.

Several limitations of the present study should be acknowledged. First, although isoflurane-based inhalation is a validated protocol for CFVR assessment in rodents, its vasodilatory and cardiodepressive effects may influence the physiological fidelity of the hyperemic response; future studies employing awake-state coronary flow imaging will be essential to validate these findings. Second, while our data support that KX enhances ITGAX transcription through a HIF-1α-dependent mechanism, further studies such as chromatin immunoprecipitation followed by quantitative PCR (ChIP-qPCR) are warranted to confirm endogenous chromatin occupancy. Finally, this study lacked validation using human samples; therefore, future translational and clinical investigations will be needed to clarify the active constituents, define the direct molecular targets, and further evaluate the clinical relevance of KX.

## 5 Conclusion

In summary, this study identifies that KX alleviates CMVD by restoring myocardial blood supply through enhanced microvascular angiogenesis and vessel stabilization. Mechanistically, KX stabilizes HIF-1α against PHD2-mediated degradation, increasing its transcriptional activity and inducing ITGAX expression. The resulting HIF-1α-ITGAX axis activates VEGF-FAK signaling, thereby promoting integrin-mediated endothelial proliferation, angiogenesis, and tight junction formation. This integrated response improves microvascular function and stabilizes nascent vascular networks, restoring myocardial blood supply and alleviating CMVD under diabetic conditions. Collectively, these findings highlight KX as a promising therapeutic candidate for diabetic microvascular complications.

## Data Availability

The raw sequencing data generated in this study have been deposited in the NCBI BioProject database under accession number PRJNA1472894 (https://www.ncbi.nlm.nih.gov/bioproject/PRJNA1472894/).
