# Supplementary material for: Kuanxiong aerosol protects against coronary microvascular dysfunction through improving angiogenesis via HIF-1α-ITGAX axis
Source: Front Pharmacol. 2026 Aug 13;17:1881934. doi: 10.3389/fphar.2026.1881934 (PMC13518324; doi:10.3389/fphar.2026.1881934)
Supplement: Supplementary file 3 [file Table1.docx]

**Table S1. Comprehensive HS-GC-MS characterization and relative quantification of KX chemical constituents.**

| **Component RT** | **Compound Name** | **Match Factor** | **Formula** | **CAS#** | **Component Area** |
| --- | --- | --- | --- | --- | --- |
| 5.838831535 | Heptane, 2,4-dimethyl- | 96.61087438 | C9H20 | 2213-23-2 | 398645.6777 |
| 7.245134594 | Benzene, 1,3-dimethyl- | 96.54167196 | C8H10 | 108-38-3 | 188880.7399 |
| 7.245192726 | o-Xylene | 95.48601043 | C8H10 | 95-47-6 | 215077.4991 |
| 7.910221653 | Isonicotinic acid, 2-phenylethyl ester | 82.71850271 | C14H13NO2 | 1000308-36-3 | 146508.0704 |
| 8.736843597 | Propanoic acid, 2-methyl-, 2-methylpropyl ester | 96.41601461 | C8H16O2 | 97-85-8 | 597843.7441 |
| 9.079363855 | alpha.-Phellandrene | 96.00674146 | C10H16 | 99-83-2 | 215037.169 |
| 9.272889517 | alpha.-Pinene | 98.98541639 | C10H16 | 80-56-8 | 5372065.88 |
| 9.737511079 | Camphene | 98.54008817 | C10H16 | 79-92-5 | 4661228.368 |
| 10.57921706 | alpha.-Phellandrene | 92.19903827 | C10H16 | 99-83-2 | 169717.055 |
| 10.65431954 | Bicyclo[3.1.1]heptane, 6,6-dimethyl-2-methylene-, (1S)- | 98.68895712 | C10H16 | 18172-67-3 | 7061708.09 |
| 11.07092696 | 5-Hepten-2-one, 6-methyl- | 97.38643387 | C8H14O | 110-93-0 | 1076094.512 |
| 11.1857549 | beta.-Myrcene | 97.55048369 | C10H16 | 123-35-3 | 679058.1712 |
| 11.57263384 | alpha.-Phellandrene | 93.28739609 | C10H16 | 99-83-2 | 1267933.01 |
| 11.58260329 | Butanoic acid, 2-methyl-, 2-methylpropyl ester | 93.77768095 | C9H18O2 | 2445-67-2 | 524246.9452 |
| 11.69889017 | Isobutyl isovalerate | 87.20452882 | C9H18O2 | 589-59-3 | 161570.8581 |
| 11.75405028 | 3-Carene | 98.40101488 | C10H16 | 13466-78-9 | 4117997.492 |
| 11.90451896 | Propanoic acid, 2-methyl-, 3-methylbutyl ester | 93.53448469 | C9H18O2 | 2050-01-3 | 275074.8709 |
| 11.97245729 | 1,3-Cyclohexadiene, 1-methyl-4-(1-methylethyl)- | 94.04463361 | C10H16 | 99-86-5 | 255913.2272 |
| 12.01604158 | Butanoic acid, 2-methylbutyl ester | 88.59068202 | C9H18O2 | 51115-64-1 | 174779.7077 |
| 12.0194284 | Propanoic acid, 2-methyl-, 3-methylbutyl ester | 87.77573998 | C9H18O2 | 2050-01-3 | 182916.3228 |
| 12.19737096 | Decane, 4-methyl- | 91.15323473 | C11H24 | 2847-72-5 | 360385.3396 |
| 12.23203259 | p-Cymene | 98.11775174 | C10H14 | 99-87-6 | 1721798.109 |
| 12.3643899 | D-Limonene | 92.35463588 | C10H16 | 5989-27-5 | 5431724.88 |
| 12.43713078 | Eucalyptol | 99.68857785 | C10H18O | 470-82-6 | 99334787.8 |
| 12.6932315 | Bicyclo[3.1.1]hept-2-ene, 3,6,6-trimethyl- | 91.33447725 | C10H16 | 4889-83-2 | 179442.7707 |
| 13.0142275 | 3-Carene | 90.62093205 | C10H16 | 13466-78-9 | 117309.5929 |
| 13.01444064 | Tricyclo[2.2.1.0(2,6)]heptane, 1,3,3-trimethyl- | 88.2158962 | C10H16 | 488-97-1 | 113362.2603 |
| 13.30430085 | Dodecane | 95.10158756 | C12H26 | 112-40-3 | 1831080.088 |
| 13.30432402 | Octane, 5-ethyl-2-methyl- | 94.89050136 | C11H24 | 62016-18-6 | 1740016.326 |
| 13.33096902 | gamma.-Terpinene | 94.96012137 | C10H16 | 99-85-4 | 551738.5519 |
| 13.47495267 | 3-Ethyl-3-methylheptane | 94.23032933 | C10H22 | 17302-01-1 | 434588.7642 |
| 13.4754482 | Undecane, 3,7-dimethyl- | 94.44576189 | C13H28 | 17301-29-0 | 518310.7793 |
| 13.75292411 | (2S)-2-(Acetyloxy)-3-methylbutanoic acid | 71.14528609 | C7H12O4 | 18667-97-5 | 142448.2569 |
| 14.25411243 | Cyclohexene, 1-methyl-4-(1-methylethylidene)- | 96.24318507 | C10H16 | 586-62-9 | 942859.9268 |
| 14.29157889 | Benzene, 4-ethenyl-1,2-dimethyl- | 88.75159421 | C10H12 | 27831-13-6 | 108823.9978 |
| 14.64004684 | Linalool | 95.05984431 | C10H18O | 78-70-6 | 1802500.554 |
| 14.69369792 | Octane, 5-ethyl-2-methyl- | 78.86023778 | C11H24 | 62016-18-6 | 488923.3039 |
| 14.69398459 | 3-Ethyl-3-methylheptane | 76.47748592 | C10H22 | 17302-01-1 | 374024.836 |
| 14.76376967 | Butanoic acid, 2-methyl-, 2-methylbutyl ester | 74.79602916 | C10H20O2 | 2445-78-5 | 175349.5138 |
| 14.86921958 | Octanoic acid, 2-tetrahydrofurylmethyl ester | 70.76845753 | C13H24O3 | 33601-75-1 | 101217.853 |
| 15.96234416 | (+)-2-Bornanone | 99.55734551 | C10H16O | 464-49-3 | 5221696.449 |
| 16.09209707 | Bicyclo[2.2.1]heptan-2-ol, 2,3,3-trimethyl- | 78.38333938 | C10H18O | 465-31-6 | 293020.0253 |
| 16.19754891 | 2,4-Cycloheptadien-1-one, 2,6,6-trimethyl- | 99.22586716 | C10H14O | 503-93-5 | 13092003.04 |
| 16.48139262 | Silane, cyclohexyldimethoxymethyl- | 91.48063572 | C9H20O2Si | 17865-32-6 | 886566.9449 |
| 16.97056583 | 3-Cyclohexen-1-ol, 4-methyl-1-(1-methylethyl)-, (R)- | 96.59670125 | C10H18O | 20126-76-5 | 5119121.533 |
| 17.21103503 | Benzenemethanol, .alpha.,.alpha.,4-trimethyl- | 71.82975572 | C10H14O | 1197-01-9 | 261016.7901 |
| 17.37714445 | alpha.-Terpineol | 96.97301141 | C10H18O | 98-55-5 | 24234882.11 |
| 17.58193711 | Succinic acid, 3-chlorophenyl 4-methoxybenzyl ester | 80.48082625 | C18H17ClO5 | 1000389-69-3 | 287065.4316 |
| 17.58816005 | Estragole | 95.947718 | C10H12O | 140-67-0 | 1392958.938 |
| 17.7533558 | Decane, 1-iodo- | 79.62172967 | C10H21I | 2050-77-3 | 374732.4503 |
| 17.90724973 | Phenol, 4-ethyl-2-methyl- | 78.70172902 | C9H12O | 2219-73-0 | 301378.5982 |
| 18.01157521 | Undecane, 2,6-dimethyl- | 87.77048369 | C13H28 | 17301-23-4 | 471076.7892 |
| 18.01788954 | Hydrogen isocyanate | 80.09522653 | CHNO | 75-13-8 | 143595.038 |
| 18.07700666 | 1,2-Benzenediol, O-(4-ethylbenzoyl)-O'-propargyloxycarbonyl- | 84.1057131 | C19H16O5 | 1000329-75-1 | 207022.19 |
| 18.21466326 | Fenchyl acetate | 91.87627104 | C12H20O2 | 13851-11-1 | 1964168.089 |
| 18.24163281 | Dodecane, 4-methyl- | 79.09831497 | C13H28 | 6117-97-1 | 449298.3643 |
| 18.62498195 | Benzene, 1-methoxy-4-methyl-2-(1-methylethyl)- | 84.795665 | C11H16O | 31574-44-4 | 210543.0091 |
| 18.87022143 | Heptane, 2,2,3,3,5,6,6-heptamethyl- | 74.9257795 | C14H30 | 7225-67-4 | 311680.0655 |
| 18.87848716 | 2-Butanone, 4-phenyl- | 92.18136343 | C10H12O | 2550-26-7 | 1794520.58 |
| 19.02636399 | Undecane, 4,7-dimethyl- | 81.35342985 | C13H28 | 17301-32-5 | 279683.0294 |
| 19.18214547 | Octane, 3,3-dimethyl- | 84.6001284 | C10H22 | 4110-44-5 | 425693.3934 |
| 19.18280049 | 3-Ethyl-3-methylheptane | 84.97732162 | C10H22 | 17302-01-1 | 436375.3069 |
| 19.42866269 | 2,4-Dimethyldodecane | 93.34462774 | C14H30 | 6117-99-3 | 1221539.397 |
| 19.51716107 | 3,5-Dimethoxytoluene | 99.47105631 | C9H12O2 | 4179-19-5 | 23669514.94 |
| 19.87750258 | Pentadecane | 94.34284776 | C15H32 | 629-62-9 | 2959076.965 |
| 20.07707119 | Bicyclo[2.2.1]heptan-2-ol, 1,7,7-trimethyl-, acetate, (1S-endo)- | 77.16269346 | C12H20O2 | 5655-61-8 | 260591.2852 |
| 20.11115762 | Nonane, 5-methyl-5-propyl- | 76.71805366 | C13H28 | 17312-75-3 | 627776.6663 |
| 20.14228946 | Safrole | 99.25391269 | C10H10O2 | 94-59-7 | 28975998.09 |
| 20.26235673 | Dodecane, 4,6-dimethyl- | 91.76688103 | C14H30 | 61141-72-8 | 586752.3464 |
| 20.41647514 | Tridecane | 97.96007971 | C13H28 | 629-50-5 | 1870601.936 |
| 20.47000831 | Decane, 3,3,6-trimethyl- | 76.10400964 | C13H28 | 62338-14-1 | 374304.8864 |
| 20.4700948 | Decane, 3,3,5-trimethyl- | 78.64633879 | C13H28 | 62338-13-0 | 385227.962 |
| 20.64957191 | Decane, 1-iodo- | 87.23301573 | C10H21I | 2050-77-3 | 236793.2673 |
| 20.64984051 | Tridecane, 5-methyl- | 86.35422232 | C14H30 | 25117-31-1 | 223287.1476 |
| 21.05608516 | Thymoquinone | 80.23136743 | C10H12O2 | 490-91-5 | 96937.09692 |
| 21.12754187 | Pentadecane | 94.45624674 | C15H32 | 629-62-9 | 1052878.981 |
| 21.50545002 | 3-((3R)-2,3-Dimethyltricyclo[2.2.1.02,6]heptan-3-yl)propanal | 95.27930432 | C12H18O | 16933-18-9 | 927328.9251 |
| 21.80783012 | Cyclohexene, 4-ethenyl-4-methyl-3-(1-methylethenyl)-1-(1-methylethyl)-, (3R-trans)- | 74.85298427 | C15H24 | 20307-84-0 | 152265.7617 |
| 21.8780646 | 3-((1S,5S,6R)-2,6-Dimethylbicyclo[3.1.1]hept-2-en-6-yl)propanal | 77.86785015 | C12H18O | 203499-08-5 | 122621.1257 |
| 22.39641357 | Ylangene | 91.10394451 | C15H24 | 14912-44-8 | 698260.8326 |
| 22.51743892 | Copaene | 94.24812551 | C15H24 | 3856-25-5 | 446093.4362 |
| 22.93059234 | Cyclohexane, 1-ethenyl-1-methyl-2,4-bis(1-methylethenyl)-, [1S-(1.alpha.,2.beta.,4.beta.)]- | 94.38923547 | C15H24 | 515-13-9 | 1318525.34 |
| 22.98351183 | Propanoic acid, 2-methyl-, 2-phenylethyl ester | 93.12219575 | C12H16O2 | 103-48-0 | 841001.4673 |
| 23.06273526 | Tetradecane | 93.30115048 | C14H30 | 629-59-4 | 433722.4141 |
| 23.2279629 | Benzene, 1,2,3-trimethoxy-5-methyl- | 85.58373597 | C10H14O3 | 6443-69-2 | 34271416.4 |
| 23.22992828 | Methyleugenol | 89.93416045 | C11H14O2 | 93-15-2 | 63493495.04 |
| 23.30783498 | 2-Decen-1-ol, (E)- | 80.20342737 | C10H20O | 18409-18-2 | 342446.2817 |
| 23.30784657 | Dodecanal | 80.47417145 | C12H24O | 112-54-9 | 380255.571 |
| 23.39666085 | Benzene, 1,2,3-trimethoxy-5-methyl- | 95.05381589 | C10H14O3 | 6443-69-2 | 10568153.76 |
| 23.51563394 | trans-.alpha.-Bergamotene | 93.70870662 | C15H24 | 13474-59-4 | 652052.9777 |
| 23.64850751 | Benzene, propoxy- | 72.33612635 | C9H12O | 622-85-5 | 1244550.924 |
| 23.65923798 | Caryophyllene | 98.31915434 | C15H24 | 87-44-5 | 16384237.19 |
| 24.03557019 | cis-.alpha.-Bergamotene | 97.84739263 | C15H24 | 18252-46-5 | 4375067.123 |
| 24.1248203 | alpha.-Guaiene | 92.52866251 | C15H24 | 3691/12/1 | 426411.8016 |
| 24.25086201 | (1R,3aS,8aS)-7-Isopropyl-1,4-dimethyl-1,2,3,3a,6,8a-hexahydroazulene | 81.48491644 | C15H24 | 36577-33-0 | 295629.4287 |
| 24.26428144 | (1,2,2-trimethyl-3-cyclopenten-1-yl)acetaldehyde | 70.65978541 | C10H16O | 1000365-94-3 | 96782.86762 |
| 24.34121556 | Bicyclo[2.2.1]heptane, 2-methyl-3-methylene-2-(4-methyl-3-pentenyl)-, (1S-endo)- | 97.67167204 | C15H24 | 25532-78-9 | 3982296.634 |
| 24.52568968 | Humulene | 97.26328645 | C15H24 | 6753-98-6 | 8566602.15 |
| 24.62819637 | 2,6,10-Trimethyltridecane | 86.8430749 | C16H34 | 3891-99-4 | 1288063.992 |
| 24.64934393 | 1,3-Benzodioxole, 4-methoxy-6-(2-propenyl)- | 81.5514322 | C11H12O3 | 607-91-0 | 1956621.832 |
| 24.66138138 | Bicyclo[2.2.1]heptane, 2-methyl-3-methylene-2-(4-methyl-3-pentenyl)-, (1S-exo)- | 94.68055982 | C15H24 | 511-59-1 | 6882685.466 |
| 24.78325866 | 3,5-Dimethyldodecane | 91.16225729 | C14H30 | 107770-99-0 | 384273.0539 |
| 24.93702909 | Dodecane, 4,6-dimethyl- | 85.387659 | C14H30 | 61141-72-8 | 320537.4429 |
| 24.937048 | Decane, 3,3,6-trimethyl- | 81.7274144 | C13H28 | 62338-14-1 | 279350.0582 |
| 25.08845061 | 1,2-Benzenediol, O-(4-butylbenzoyl)-O'-(butoxycarbonyl)- | 72.6271671 | C22H26O5 | 1000329-73-7 | 272779.7225 |
| 25.0943787 | 1-Pentadecene | 94.95453985 | C15H30 | 13360-61-7 | 2442136.388 |
| 25.20768111 | Germacrene D | 90.05390399 | C15H24 | 23986-74-5 | 6504457.211 |
| 25.24436961 | Pentadecafluorooctanoic acid, pentadecyl ester | 88.48883184 | C23H31F15O2 | 1000406-04-5 | 5495143.355 |
| 25.25441782 | N-Hydroxypropanamide, O-acetyl derivative | 74.04287464 | C5H9NO3 | 1000521-27-9 | 699854.2222 |
| 25.32790991 | 3-Ethyl-3-methylheptane | 76.77260756 | C10H22 | 17302-01-1 | 968727.6946 |
| 25.34512724 | Naphthalene, decahydro-4a-methyl-1-methylene-7-(1-methylethenyl)-, [4aR-(4a.alpha.,7.alpha.,8a.beta.)]- | 96.85970549 | C15H24 | 17066-67-0 | 2887428.683 |
| 25.47024865 | Hexadecane | 87.21696497 | C16H34 | 544-76-3 | 3355071.006 |
| 25.47032409 | Dodecane, 4,6-dimethyl- | 87.19024707 | C14H30 | 61141-72-8 | 3332880.376 |
| 25.53843023 | Asaricin | 84.91727076 | C11H12O3 | 18607-93-7 | 8392172.888 |
| 25.56046924 | Pentadecane | 88.72853766 | C15H32 | 629-62-9 | 9488202.649 |
| 25.68236667 | cis-alpha-Bisabolene | 93.60608479 | C15H24 | 29837-07-8 | 1566990.373 |
| 25.69364159 | Undecane, 3,5-dimethyl- | 70.19207299 | C13H28 | 17312-81-1 | 380530.4994 |
| 25.83648477 | beta.-Bisabolene | 97.94037097 | C15H24 | 495-61-4 | 7982622.767 |
| 25.90166528 | 2,4-Di-tert-butylphenol | 91.20417507 | C14H22O | 96-76-4 | 6468091.446 |
| 25.91537406 | Cubenene | 74.36018929 | C15H24 | 29837-12-5 | 1254166.789 |
| 25.93647077 | Butylated Hydroxytoluene | 74.7208997 | C15H24O | 128-37-0 | 809628.7353 |
| 26.00943387 | Naphthalene, 1,2,3,4,4a,5,6,8a-octahydro-7-methyl-4-methylene-1-(1-methylethyl)-, (1.alpha.,4a.beta.,8a.alpha.)- | 98.84816887 | C15H24 | 39029-41-9 | 5598140.754 |
| 26.111153 | (2S,4aR,8aR)-4a,8-Dimethyl-2-(prop-1-en-2-yl)-1,2,3,4,4a,5,6,8a-octahydronaphthalene | 89.53927158 | C15H24 | 123123-37-5 | 1206366.474 |
| 26.14455597 | Decane, 3-ethyl-3-methyl- | 70.14913853 | C13H28 | 17312-66-2 | 247799.6385 |
| 26.21493249 | Naphthalene, 1,2,3,5,6,8a-hexahydro-4,7-dimethyl-1-(1-methylethyl)-, (1S-cis)- | 94.07277586 | C15H24 | 483-76-1 | 2613893.605 |
| 26.40314772 | (E)-1-Methyl-4-(6-methylhept-5-en-2-ylidene)cyclohex-1-ene | 90.66069057 | C15H24 | 53585-13-0 | 1395995.071 |
| 26.51692698 | Naphthalene, 1,2,3,4,4a,5,6,8a-octahydro-7-methyl-4-methylene-1-(1-methylethyl)-, (1.alpha.,4a.beta.,8a.alpha.)- | 71.35414264 | C15H24 | 39029-41-9 | 290198.3553 |
| 26.54687936 | Hexadecane | 90.90959484 | C16H34 | 544-76-3 | 1639704.52 |
| 26.64340959 | Cyclohexene, 4-[(1E)-1,5-dimethyl-1,4-hexadien-1-yl]-1-methyl- | 96.32079686 | C15H24 | 25532-79-0 | 2631255.325 |
| 26.69860548 | alpha.-Calacorene | 94.60644388 | C15H20 | 21391-99-1 | 540182.2181 |
| 26.7838968 | Dodecane, 1-iodo- | 81.91287408 | C12H25I | 4292-19-7 | 276272.7779 |
| 26.94844124 | Benzene, 1,2,3-trimethoxy-5-(2-propenyl)- | 98.33346529 | C12H16O3 | 487-11-6 | 5709101.04 |
| 27.04512344 | (1S,2E,6E,10R)-3,7,11,11-Tetramethylbicyclo[8.1.0]undeca-2,6-diene | 86.30961319 | C15H24 | 24703-35-3 | 459569.228 |
| 27.15531037 | Benzenemethanol, 4-methyl-.alpha.-(1-methyl-2-propenyl)-, (R*,R*)- | 86.22637379 | C12H16O | 83173-76-6 | 1228395.082 |
| 27.15582904 | gamma.-Elemene | 85.43451588 | C15H24 | 29873-99-2 | 1268224.219 |
| 27.36298711 | Benzene, 1,2,3-trimethoxy-5-(1-propenyl)-, (E)- | 83.4845565 | C12H16O3 | 5273-85-8 | 324091.9638 |
| 27.66397652 | Caryophyllene oxide | 96.39911081 | C15H24O | 1139-30-6 | 4458416.641 |
| 27.77645086 | Selina-3,7(11)-diene | 83.49762342 | C15H24 | 6813-21-4 | 435694.1798 |
| 27.77670852 | (2S,4aR,8aR)-4a,8-Dimethyl-2-(prop-1-en-2-yl)-1,2,3,4,4a,5,6,8a-octahydronaphthalene | 79.85062064 | C15H24 | 123123-37-5 | 444040.2537 |
| 27.89217856 | Benzenemethanol, 4-methyl-.alpha.-(1-methyl-2-propenyl)-, (R*,R*)- | 71.38405709 | C12H16O | 83173-76-6 | 412560.3841 |
| 27.9213782 | Hexadecane | 83.35753079 | C16H34 | 544-76-3 | 318542.0852 |
| 28.27769812 | (1R,3E,7E,11R)-1,5,5,8-Tetramethyl-12-oxabicyclo[9.1.0]dodeca-3,7-diene | 94.78745726 | C15H24O | 19888-34-7 | 1592577.524 |
| 28.40561475 | 4a(2H)-Naphthalenol, 1,3,4,5,6,8a-hexahydro-4,7-dimethyl-1-(1-methylethyl)-, (1S,4S,4aS,8aR)- | 89.11230276 | C15H26O | 73365-77-2 | 1089452.665 |
| 28.73171508 | Hexadecane | 89.61341856 | C16H34 | 544-76-3 | 861721.3447 |
| 28.7322529 | Heptadecane | 89.58688543 | C17H36 | 629-78-7 | 872666.6129 |
| 28.8077119 | 2-Hydroxy-4,5-methylenedioxypropiophenone | 91.48645516 | C10H10O4 | 18607-90-4 | 1123652.714 |
| 28.89361714 | Cyclopentanone, 2,5-dicyclopentylidene- | 72.34411342 | C15H20O | 5682-82-6 | 722999.4372 |
| 29.30409696 | Cyclobuta[1,2:3,4]dicyclooctene, 1,2,5,6,6a,6b,7,8,11,12,12a,12b-dodecahydro-, (6a.alpha.,6b.alpha.,12a.beta.,12b.beta.)- | 84.52304175 | C16H24 | 61233-68-9 | 4360662.015 |
| 29.63961309 | 3-Cyclohexen-1-ol, 1-[(1S)-1,5-dimethyl-4-hexenyl]-4-methyl-, (1S)- | 82.37825299 | C15H26O | 15352-77-9 | 3696754.41 |
| 29.71098381 | 8-Heptadecene | 79.27968957 | C17H34 | 2579/4/6 | 4401767.048 |
| 29.81749131 | (E)-5-((1R,3R,6S)-2,3-Dimethyltricyclo[2.2.1.02,6]heptan-3-yl)-2-methylpent-2-enal | 92.90970244 | C15H22O | 19903-70-9 | 12114263.42 |
| 29.89593568 | 3-Heptadecene, (Z)- | 97.08191875 | C17H34 | 1000141-67-3 | 7210483.277 |
| 30.1044257 | beta.-Santalol | 82.80780638 | C15H24O | 77-42-9 | 3221974.23 |
| 30.172812 | Heptadecane | 98.79882443 | C17H36 | 629-78-7 | 7147815.93 |
| 30.31415846 | Dodecane, 4,6-dimethyl- | 73.32985098 | C14H30 | 61141-72-8 | 661773.3585 |
| 30.32182315 | Decane, 3,3,8-trimethyl- | 74.59901855 | C13H28 | 62338-16-3 | 629736.8448 |
| 30.40689935 | Heptacosane | 87.27264909 | C27H56 | 593-49-7 | 4020881.802 |
| 30.74277388 | 2-Penten-1-ol, 2-methyl-5-(2-methyl-3-methylenebicyclo[2.2.1]hept-2-yl)-, [1S-[1.alpha.,2.alpha.(E),4.alpha.]]- | 77.44037797 | C15H24O | 37172-32-0 | 10682906.43 |
| 31.12539688 | Bromonitromethane | 72.24019766 | CH2BrNO2 | 563-70-2 | 68656149.92 |
| 31.33640502 | Heneicosane | 80.37813097 | C21H44 | 629-94-7 | 1505334.821 |
| 31.33665248 | Hexadecane, 2,6,10,14-tetramethyl- | 79.24373886 | C20H42 | 638-36-8 | 6080383.027 |
| 31.36333461 | L-Alanine, N-benzoyl-, methyl ester | 71.58091466 | C11H13NO3 | 7244-67-9 | 2102781.518 |
| 32.25869038 | alpha.-Furil | 78.30609604 | C10H6O4 | 492-94-4 | 124080.1749 |
| 32.41076441 | Carbonic acid, butyl phenyl ester | 77.44600993 | C11H14O3 | 4824-76-4 | 21470469.26 |
| 33.29577517 | Octadecane | 83.13945782 | C18H38 | 593-45-3 | 1168283.697 |
| 33.90127104 | 9-Nonadecene | 82.7317395 | C19H38 | 31035-07-1 | 1052134.376 |
| 34.09113864 | Eicosyl octyl ether | 91.86454374 | C28H58O | 1000406-38-8 | 1769338.201 |
| 34.10035482 | Cyclobutaneacetonitrile, 1-methyl-2-(1-methylethenyl)- | 73.05977562 | C10H15N | 55760-15-1 | 513553.7996 |
| 34.10041103 | Hex-4-yn-3-one | 70.08541827 | C6H8O | 10575-41-4 | 487365.9059 |
| 34.15622578 | Hexadecane | 76.01736574 | C16H34 | 544-76-3 | 974081.3757 |
| 34.32475917 | Nonadecane | 91.07104985 | C19H40 | 629-92-5 | 1001514.888 |
| 34.6768265 | Undecane, 3,9-dimethyl- | 78.99523211 | C13H28 | 17301-31-4 | 328759.2776 |
| 34.67723107 | Dodecane, 1-iodo- | 81.99856984 | C12H25I | 4292-19-7 | 369736.8295 |
| 34.81434425 | Heneicosane | 91.38283654 | C21H44 | 629-94-7 | 2835787.45 |
| 34.96309158 | 5H-Tetrazol-5-amine | 71.99433168 | CH3N5 | 1000273-02-0 | 444523.6947 |
| 35.21633687 | Benzenepropanoic acid, 3,5-bis(1,1-dimethylethyl)-4-hydroxy-, methyl ester | 78.85123301 | C18H28O3 | 6386-38-5 | 120721.4803 |
| 35.51205887 | n-Hexadecanoic acid | 83.02965689 | C16H32O2 | 1957/10/3 | 598982.3321 |
| 35.63729041 | Heneicosane | 90.31712526 | C21H44 | 629-94-7 | 1501422.186 |
| 37.41586745 | Heneicosane | 84.45479234 | C21H44 | 629-94-7 | 552047.2325 |
| 37.41598023 | Octadecane | 83.78986721 | C18H38 | 593-45-3 | 480224.2295 |
| 38.11928032 | 3-Ethyl-2,6,10-trimethylundecane | 84.73060127 | C16H34 | 1000432-25-9 | 350635.5138 |
| 38.61847559 | Heneicosane | 84.07236284 | C21H44 | 629-94-7 | 710213.649 |
| 38.82326518 | Heptadecane, 2-methyl- | 75.33342192 | C18H38 | 1560-89-0 | 135887.2484 |
| 39.39860128 | Heptadecane | 76.15944177 | C17H36 | 629-78-7 | 373879.6389 |
| 39.39904296 | Dodecane, 4,6-dimethyl- | 76.11118481 | C14H30 | 61141-72-8 | 368052.9943 |
| 39.55132003 | Hexadecane, 2,6,11,15-tetramethyl- | 89.88468285 | C20H42 | 504-44-9 | 950965.6775 |
| 39.55162086 | Hexadecane, 2,6,10,14-tetramethyl- | 91.09160503 | C20H42 | 638-36-8 | 985513.6076 |
| 41.67134754 | 1,7-Diphenyl-4-hepten-3-one | 90.67880427 | C19H20O | 79559-59-4 | 671125.3571 |
| 42.46338359 | Triacontane, 1-iodo- | 85.09790889 | C30H61I | 1000406-32-3 | 743852.5393 |
| 42.46345404 | Dotriacontane, 1-iodo- | 84.08772198 | C32H65I | 1000406-32-4 | 786804.7963 |
